# Enlarged colitogenic T cell population paradoxically supports colitis prevention through the B-lymphocyte-dependent peripheral generation of CD4^+^Foxp3^+^ Treg cells

**DOI:** 10.1038/srep28573

**Published:** 2016-06-29

**Authors:** Fábio Barrozo do Canto, Sylvia Maria Nicolau Campos, Alessandra Granato, Rafael F. da Silva, Luciana Souza de Paiva, Alberto Nóbrega, Maria Bellio, Rita Fucs

**Affiliations:** 1 Universidade Federal do Rio de Janeiro (UFRJ), Centro de Ciências da Saúde, Instituto de Microbiologia Paulo de Góes (IMPG), Departamento de Imunologia, Laboratório Integrado de Imunobiologia - Rio de Janeiro/RJ, Brazil; 2 Universidade Federal Fluminense (UFF), Instituto de Biologia, Departamento de Imunobiologia, Campus do Valonguinho, Outeiro de São João Batista, s/n. Niterói, RJ 24210-130 - Brazil; 3 Grupo de Imunologia Gastrintestinal – UFF, Campus do Valonguinho, Outeiro de São João Batista, s/n. Niterói, RJ 24210-130 - Brazil; 4 Programa de Pós-Graduação em Patologia - Faculdade de Medicina/UFF, Campus do Valonguinho, Outeiro de São João Batista, s/n. Niterói, RJ 24210-130 - Brazil; 5 Laboratório de Patologia Celular e Molecular – UFF, Campus do Valonguinho, Outeiro de São João Batista, s/n. Niterói, RJ 24210-130 - Brazil; 6 Programa de Pós-Graduação em Biologia das Interações - Instituto de Biologia/UFF, Campus do Valonguinho, Outeiro de São João Batista, s/n. Niterói, RJ 24210-130 - Brazil; 7 Núcleo de Animais de Laboratório, Laboratório de Imunorregulação – UFF, Campus do Valonguinho, Outeiro de São João Batista, s/n. Niterói, RJ 24210-130 - Brazil.

## Abstract

Intestinal inflammation can be induced by the reconstitution of T/B cell-deficient mice with low numbers of CD4^+^ T lymphocytes depleted of CD25^+^Foxp3^+^ regulatory T cells (Treg). Using RAG-knockout mice as recipients of either splenocytes exclusively depleted of CD25^+^ cells or FACS-purified CD4^+^CD25^−^Foxp3^−^ T cells, we found that the augmentation of potentially colitogenic naïve T cell numbers in the inoculum was unexpectedly beneficial for the suppression of colon disease and maintenance of immune homeostasis. Protection against T cell-mediated colitis correlated with a significant increment in the frequency of peripherally-induced CD4^+^CD25^+^Foxp3^+^ T (pTreg) cells, especially in the mesenteric lymph nodes, an effect that required the presence of B cells and CD4^+^CD25^−^Foxp3^+^ cells in physiological proportions. Our findings support a model whereby the interplay between B lymphocytes and a diversified naïve T cell repertoire is critical for the generation of CD4^+^CD25^+^Foxp3^+^ pTreg cells and colitis suppression.

The experimental induction of colitis by the adoptive transfer of naïve T cells into lymphopenic recipients has been extensively demonstrated^1^ and CD4^+^ T lymphocytes were shown to constitute the main cell population mediating colonic inflammation^2^. Initially described as CD4^+^CD45RB^high^ cells^3^, the colitogenic CD4^+^ subset was later characterized as CD25^−^Foxp3^− ^4. Regulatory T cells (Treg), both necessary and sufficient to prevent colonic inflammation, are predominantly present within the CD4^+^CD45RB^low^ fraction^5^ and constitutively express CD25 and Foxp3. This subset constitutes approximately 5–15% of the peripheral CD4^+^ T lymphocytes and comprises both thymus-emigrated Treg cells (tTregs) and peripherally derived-Treg cells (pTregs)^6^.

It is generally accepted that the repertoire of tTreg cell specificities is self-antigen-biased, since intra-thymic Treg differentiation requires high-affinity interactions with MHC:self-peptides^7^,^8^,^9^, while Foxp3^+^ pTregs, which develop in the post-thymic compartment from Foxp3^−^ naïve T cells, may include a broader range of specificities, predominantly towards non-self peptides^10^. It was recently shown that pTregs are indispensable for the control of colitis^11^ and autoimmune responses^12^. It is believed that, by complementing each other’s TCR repertoires, pTregs and tTregs collaborate for the suppression of autoimmune and inflammatory diseases^13^.

The finding that pTregs are indispensable for the control of colitis raises important questions. How are pTregs generated from CD4^+^CD25^−^Foxp3^−^ T cells? What are the critical cell types participating in this process? Does the diversity of CD4^+^CD25^−^Foxp3^−^ T cell repertoire affect the emergence of pTregs? Specifically concerning this last point, one could hypothesize that the numerical enlargement of the naïve CD4^+^CD25^−^ T cell pool transferred to lymphopenic recipients might be paradoxically beneficial for the suppression of colitis, as the source of relevant clones available for peripheral conversion to Foxp3^+^ cells would be also presumably broadened.

In fact, low numbers of purified colitogenic CD4^+^CD45RB^hi^ T cells (0.4–1.0 × 10^6^) are normally used to induce lethal colitis in T/B cell-deficient recipients^14^. Limited pTreg conversion from this very constrained source of conventional T cells has been reported^15^,^16^ and could be put forward as an important factor to explain the magnitude of colon inflammation induced by a reduced CD4^+^CD25^−^ T cell inoculum. Noteworthy, it was reported that augmentation of the inoculated naïve purified T cell pool (up to 10 × 10^6^ Treg-depleted CD4^+^CD45RB^hi^ cells) does not lead to colitis prevention^17^. Although pTreg cell generation was not addressed in such condition, this phenomenon was probably insufficient to mediate intestinal homeostasis, as mice receiving low and high doses of colitogenic CD4^+^ T cells displayed equivalent colon disease. This has been taken as evidence that tTreg deprivation, and not defective pTreg generation, is the crucial requirement for unleashing intestinal inflammation.

The failure to afford colitis protection using larger numbers of naïve CD4^+^ T cells could, alternatively, be secondary to the lack of relevant immune cell types required to expand Treg cell numbers *in vivo*. One marked feature of the colitis induction by adoptive T-cell transfer is the high purity of inoculated cells, which often exceeds 95% of conventional CD4^+^CD25^−^CD45RB^high^ cells. The lymphopenia-driven expansion of these lymphocytes, therefore, takes place in the absence of several adaptive immune cell types (such as CD8^+^/γδ T cells and B lymphocytes), which have been depleted from the inoculum and are not endogenously produced by T/B cell-deficient hosts. It has become evident that the peripheral numbers of Treg cells can be modulated by antigen-presenting cells (APCs), such as dendritic cells^18^ and B lymphocytes^19^,^20^. However, it has never been determined whether, in the presence of physiological proportions of non-T cells in the inoculum, increased numbers of naïve CD4^+^ T cells could generate enough pTreg to influence the outcome of colon disease.

In order to address this question, we transferred large numbers of total splenocytes, exclusively depleted of CD25^+^ Treg cells, into Rag^−/−^ mice. Using different doses of this inoculum, which contains physiological levels of all non-T cell splenic populations but Treg cells, we showed that the augmentation of potentially colitogenic CD4^+^CD25^−^ cells inoculated leads to suppression of colitis. The protection was associated with a B cell-dependent increase in the systemic CD4^+^CD25^+^Foxp3^+^ Treg cell frequency, an effect observed only when the initial CD4^+^CD25^−^ T cell fraction co-inoculated was also augmented. The presence of CD4^+^CD25^−^Foxp3^+^ T cells was essential to the recovery of peripheral Treg cell numbers and disease prevention. Importantly, the emergence of CD25^+^Foxp3^+^ pTregs was significantly impaired when the initial size of the CD4^+^CD25^−^Foxp3^−^ precursors was reduced, which correlated with disease induction. Our results raise the notion that the numerical enlargement of the conventional CD4^+^CD25^−^Foxp3^−^ T cell pool, presumably bringing higher diversification of T cell repertoire in parallel, may be beneficial for colitis suppression as a consequence of improved B cell-driven peripheral conversion to CD4^+^Foxp3^+^ regulatory T cells. The implications of these findings for clinical therapies currently used to control autoimmune diseases are discussed.

## Results

### An augmented number of potentially colitogenic cells unexpectedly prevents T cell-mediated colitis when transferred to lymphopenic hosts

**Figure 1:**
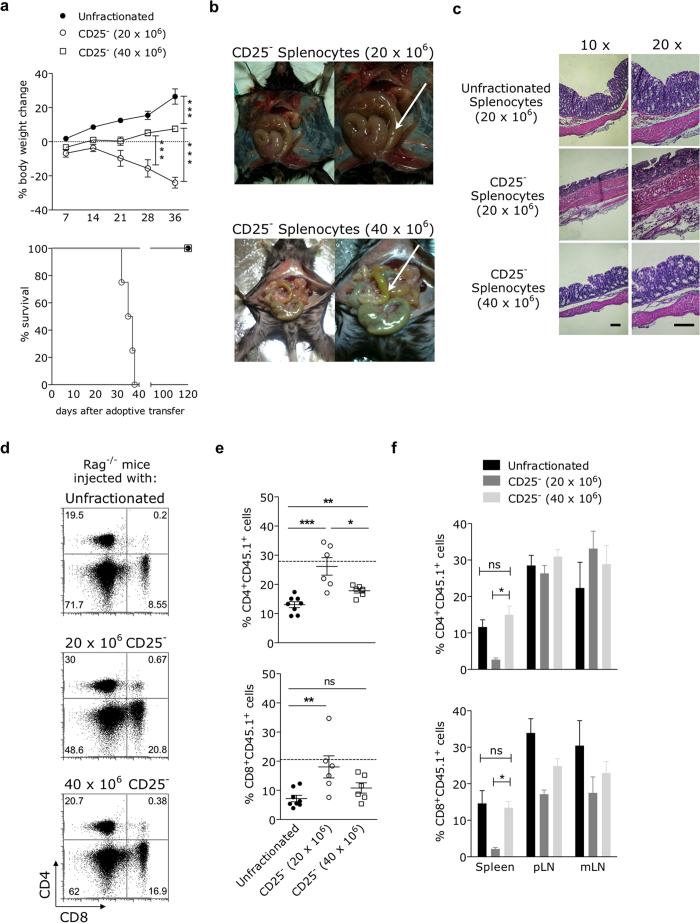
T cell-mediated colitis is surprisingly prevented following lymphopenic expansion of an augmented number of CD25^−^ cells. B6.*Rag2*^−/−^ mice were adoptively transferred with either unfractionated splenocytes or splenocytes depleted of CD25^+^ cells (20 or 40 × 10^6^ cells) isolated from adult CD45.1^+^ C57BL/6.SJL donors. **(a)** Mice were monitored for body weight changes (left) and survival (right) every three days after adoptive transfer. **(b,c)** Mice injected with CD25^−^ splenocytes were euthanized four weeks after adoptive transfer and colons were analyzed **(b)** macroscopically and **(c)** histologically (colon sections were stained for H & E; horizontal black bars = 100 μm; magnifications are indicated above panels). **(d,e)** Blood samples were collected 14 days after adoptive transfer and the frequencies of donor CD4^+^CD45.1^+^ and CD8^+^CD45.1^+^ T cells were determined by flow cytometry. **(d)** Dot-plots representative of one animal within each experimental group. Numbers plotted in each quadrant refer to the percentage of events accumulated on lymphocyte gate established on the basis of FSC × SSC parameters. **(e)** Data pooled from two independent experiments (with at least three mice per group, mean ± SEM) are shown. Average T cell frequencies found in euthymic B6 mice are included for comparison (dashed horizontal lines). **(f)** Spleens, peripheral lymph nodes (pooled axillary, inguinal and brachial LNs) and mesenteric lymph nodes were harvested 30 days after adoptive transfer and donor-derived T cells frequencies were determined by flow cytometry. Bar graphs show mean values ± SEM of peripheral donor T cell frequencies from at least three mice individually tested per group. *p < 0.05, **p < 0.01, ***p < 0.001; not significant (ns): p > 0.05.

Our primary interest was to verify if the absolute number of conventional CD4^+^CD25^−^ T cells transferred to lymphopenic recipients could influence the development of colonic inflammation when associated with proportional levels of non-T cells. For that, we adoptively transferred *Rag2*^−/−^ mice with two different doses of CD25^+^ cell-depleted splenocytes (20 × 10^6^ and 40 × 10^6^ cells, containing about 3 and 6 × 10^6^ mature CD4^+^CD25^−^ T cells, respectively) and the clinical and histological signs of colitis were monitored. The purity and phenotype of transferred cells are shown in Supplementary Figure 1. Mice injected with the low dose of CD25^−^ splenocytes progressively lost weight (Fig. 1a, top), culminating with the death of the majority of animals 4–5 weeks after transfer (Fig. 1a, bottom). In contrast, mice injected with the high dose of potentially colitogenic cells unexpectedly gained weight and survived indefinitely, remaining as healthy as control mice reconstituted with unfractionated splenocytes.

As expected, mice injected with the low dose of CD25^−^ splenocytes showed a significant enlargement of the colon four weeks after adoptive transfer (Fig. 1b, upper panel), which was associated with extensive destruction of mucosal layer, absence of mucin-secreting globet cells and crypt erosion (Fig. 1c, middle panel). Strikingly, those signs were completely absent from the colonic tissue of high dose-injected mice, whose colons were macro (Fig. 1b, bottom panel) and microscopically similar to those of controls injected with unfractionated splenocytes (Fig. 1c, upper and bottom panels). Consistent with that, the high dose-injected hosts, although inoculated with a two-fold higher absolute number of CD25^−^ T cells, showed CD4^+^ and CD8^+^ T cell frequencies lower than those seen in low dose-injected counterparts (Fig. 1d,e).

The percentages of CD4^+^ and CD8^+^ T cells were also determined in secondary lymphoid organs 30 days after the injection of CD25^−^ splenocytes. We found an expressive decrease in the frequencies of CD4^+^ and CD8^+^ T lymphocytes in the spleen, but not in lymph nodes, of hosts transferred with the low dose of CD25^−^ splenic cells, when compared to high dose-injected counterparts. Notably, mice receiving CD25^−^ high-dose inoculum displayed splenic T cell levels that resembled those found in hosts of unfractionated splenocytes (Fig. 1f), showing that the injection of a two-fold augmented number of CD25^−^ cells allowed the establishment of T cell frequencies closer to those observed in healthy controls.

All the high dose-injected recipients were followed for at least 8 months after adoptive transfer and none of them died or showed any clinical sign of disease (not shown). This prolonged survival, equivalent to that observed in counterparts receiving unfractionated splenocytes, was consistent with the preservation of colonic mucosa. Therefore, the increase of transferred CD25^−^ cell numbers resulted in long-term suppression of colitis development.

### Prevention of T cell-mediated colitis correlates with a higher frequency of CD4^+^Foxp3^+^ regulatory T cells

**Figure 2:**
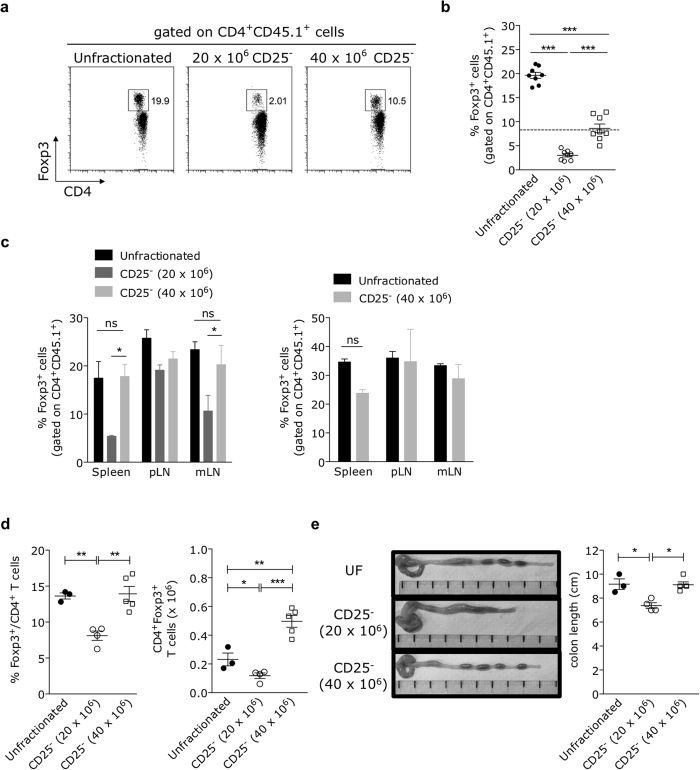
Prevention of colitis correlates with an increased peripheral frequency of CD4^+^Foxp3^+^ T cells. **(a,b)** Blood samples of mice described in Fig. 1 were collected 28 days after adoptive transfer and the frequencies of Foxp3^+^ cells, gated on CD4^+^CD45.1^+^, were determined by flow cytometry. **(a)** Dot-plots representative of one animal within each experimental group. **(b)** Data pooled from two independent experiments (with at least three mice per group, mean ± SEM) are shown. Average Foxp3^+^ T cell frequencies found in euthymic B6 mice are included for comparison (dashed horizontal line). **(c)** Foxp3^+^ cell frequencies, relative to total CD4^+^ T cells, were determined in spleens, peripheral lymph nodes (pooled axillary, inguinal and brachial LNs) and mesenteric lymph nodes at 1 month (left) or 8 months (right) after reconstitution. Bar graphs show mean ± SEM of Foxp3^+^ cells (gated on total CD4^+^ T cells) from at least 3 mice individually tested per group. **(d,e)** Frequencies (D, left) and absolute numbers (D, right) of CD4^+^Foxp3^+^ cells were determined in the spleen of reconstituted mice 28 days after injection. Colon length (**e**) was determined for each experimental condition and representative photographs of the organ are shown per group. Data are representative of two independent experiments with similar results. *p < 0.05, **p < 0.01, ***p < 0.001; not significant (ns): p > 0.05.

We next hypothesized that the protection against colitis observed in mice reconstituted with the high dose of CD25^−^ splenocytes might be due to an increase of peripheral CD4^+^Foxp3^+^ regulatory T cell proportions. One month after transfer, the expansion of unfractionated splenocytes under lymphopenic settings resulted in circulating CD4^+^Foxp3^+^ T cell frequencies higher than those usually found in lympho-replete euthymic mice (Fig. 2a,b), a result we had previously obtained^21^. Mice injected with the lower dose of CD25^−^ splenocytes showed significantly reduced levels of circulating CD4^+^Foxp3^+^ T cells (average of 3.5%), while hosts injected with the higher dose of the same inoculum generated a robust rise in the frequency of that subset (average of 8.5%) (Fig. 2a,b), reaching numbers similar to those observed in lympho-replete euthymic mice.

The selective rise in Foxp3^+^ cell frequencies within circulating CD4^+^ T cells in recipients of the high-dose CD25^−^ splenocytes was also evident in spleen and mesenteric lymph nodes, with percentages indistinguishable from those observed in mice inoculated with unfractionated splenocytes (Fig. 2c, left). In comparison, low dose-injected mice displayed significant lower levels of the regulatory CD4^+^ subset in the same peripheral organs (Fig. 2c, left). Accordingly, animals injected with the high-dose of CD25^−^ splenocytes showed a significantly higher absolute number of splenic CD4^+^Foxp3^+^ Treg cells relative to low dose-injected recipients (Fig. 2d). The increase of splenic Treg cell numbers in high dose-injected mice correlated with a normal colon length, similar to that observed in controls reconstituted with unfractionated splenocytes (Fig. 2e). The augmented frequency of Treg cells was kept stable in recipients of the high-dose CD25^−^ splenocytes for at least 8 months after the adoptive transfer (Fig. 2c, right). As mice injected with either the low or the high dose of CD25^+^ cell-depleted splenocytes received the same relative proportions of all splenic subpopulations and the absolute number of Foxp3^+^ Treg cell contaminants was very low (Supplementary Figure 1), our results suggest that the numerical enlargement of CD25^−^ T cell pool in the inoculum may have allowed the expansion of CD25^−^Foxp3^+^ Treg cell contaminants and/or peripheral conversion of conventional CD4^+^CD25^−^Foxp3^−^ T cells to CD4^+^Foxp3^+^ T cells at a level enough to suppress colonic inflammation.

### The rise in Foxp3^+^ T cell frequencies is not observed if the CD25^−^ high-dose inoculum is depleted of non-CD4^+^ T cells

**Figure 3:**
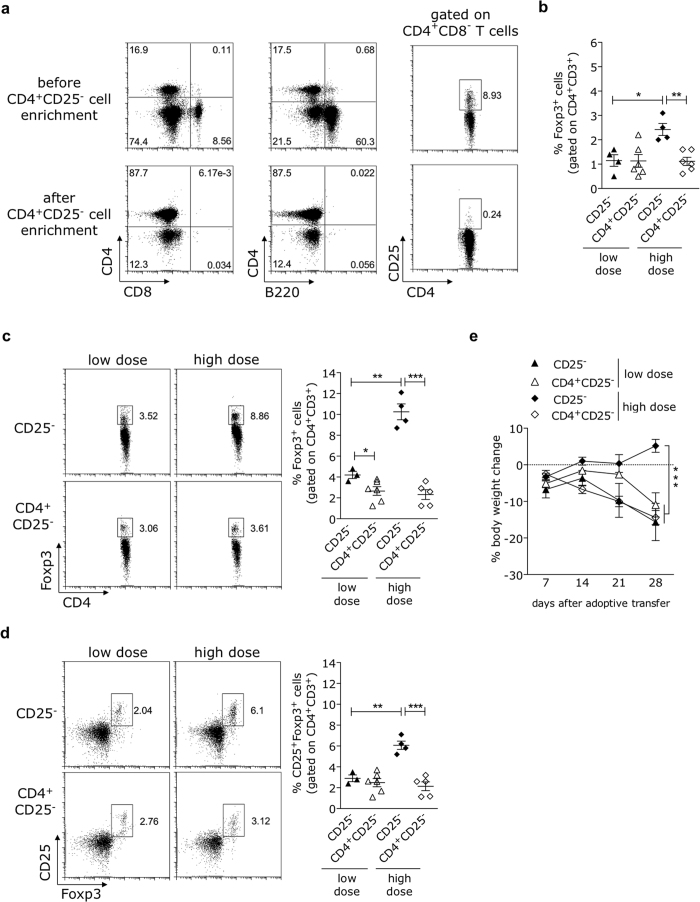
Lymphopenic expansion of the high dose of CD25^−^CD4^+^-enriched splenocytes does not result in increased Foxp3^+^ regulatory T cell frequencies. B6.*Rag2*^−/−^ mice were adoptively transferred with either 3 or 6 × 10^6^ CD4^+^-enriched splenocytes depleted of CD25^+^ cells (CD4^+^CD25^−^) isolated from adult C57BL/6 donors, absolute numbers of CD4^+^CD25^−^ equivalent to those present in low (20 × 10^6^) and high (40 × 10^6^) doses of CD25^−^ splenocytes (here referred to as CD25^−^), respectively. (**a**) Representative flow cytometry dot-plots show the relative enrichment of splenic CD4^+^CD25^−^ cells by MACS-based negative selection. Splenocytes enriched in CD4^+^ T cells were 87,5-92% pure and included no B220- or CD8-expressing cells. (**b**–**d**) The relative frequencies of Foxp3^+^ and CD25^+^Foxp3^+^ T cells (gated on CD4^+^ T cells) were determined in blood by flow cytometry at days 14 (**b**) and 28 (**c,d**) after injection. (**e**) Mice were monitored for body weight changes (determined as the percentage of variation relative to the initial weight value) every week after adoptive transfer. Data representative of two independent experiments (mean ± SEM) with at least 3 mice per group are shown. *p < 0.05, **p < 0.01, ***p < 0.001; not significant (ns): p > 0.05.

In order to determine whether the increase in the peripheral Treg cell frequency seen in recipients of the high-dose CD25^−^ splenocytes requires the presence of non-CD4^+^ cell types, we adoptively transferred *Rag2*^−/−^ mice with low and high doses of CD25^−^ splenocytes also depleted of non-CD4^+^ T cells (B220^+^, CD8α^+^, γδ^+^, CD11b^+^, CD11c^+^ and CD49d^+^), thereby resulting in a population highly enriched for CD4^+^CD25^−^ T cells (hereafter referred to as CD4^+^CD25^−^-enriched splenocytes) (Fig. 3a). Mice receiving low and high doses of CD25^+^ cell-depleted splenocytes were included as control groups.

As shown in Fig. 3b, the elevation of Treg cell frequencies is already detectable two weeks after reconstitution with the high dose of CD25^−^ splenocytes. Elimination of the major non-CD4^+^ splenic cell types significantly impacted the peripheral recovery of Treg cell frequencies in recipients of the high-dose inoculum, but had no effect on the numbers of Treg cells in mice receiving the low dose of CD4^+^CD25^−^ cells. The two-fold increase of Foxp3^+^ (Fig. 3b) or CD25^+^Foxp3^+^ (not shown) levels in the blood of hosts receiving the high-dose CD25^−^ splenocytes was abrogated by the elimination of non-CD4^+^ cells from the inoculum, resulting in percentages equivalent to those found in recipients adoptively transferred with low doses of CD25^−^ or CD4^+^CD25^−^-enriched splenocytes. Noteworthy, the inability to recover the Foxp3^+^ T cell compartment was stable over time in recipients of CD4^+^CD25^−^ T cells, contrasting with the gradual growth of Foxp3^+^ T cell numbers observed 28 days after reconstitution with the high dose of CD25^−^ splenocytes (Fig. 3c,d). Importantly, while all mice receiving CD4^+^CD25^−^-enriched splenocytes lost weight (at a similar rate as recipients of the low dose of CD25^−^ splenocytes), animals injected with the high dose of CD25^−^ splenocytes gained weight and remained healthy over the time course studied. These data showed, therefore, that non-CD4^+^ cells present in the inoculum are required to promote the augmentation of CD4^+^Foxp3^+^ T cell proportions in recipients of the high number of CD25^−^ splenocytes, a step relevant for preventing intestinal inflammation.

### B lymphocytes are required for the increase of Treg cell frequencies upon peripheral expansion of CD25^−^ splenocytes

We next wanted to identify which non-CD4^+^ cell type present in the inoculum of CD25^−^ splenocytes was necessary to allow for the numerical increase of Treg cells following reconstitution with high numbers of CD4^+^CD25^−^ cells. As B lymphocytes constitute the major cell type among non-CD4^+^ splenic cells and are not endogenously produced by Rag-deficient mice, we assessed whether this lymphoid cell type might be involved by injecting *Rag2*^−/−^ hosts with B cell-depleted CD25^−^ splenocytes (Supplementary Figure 2A).

**Figure 4:**
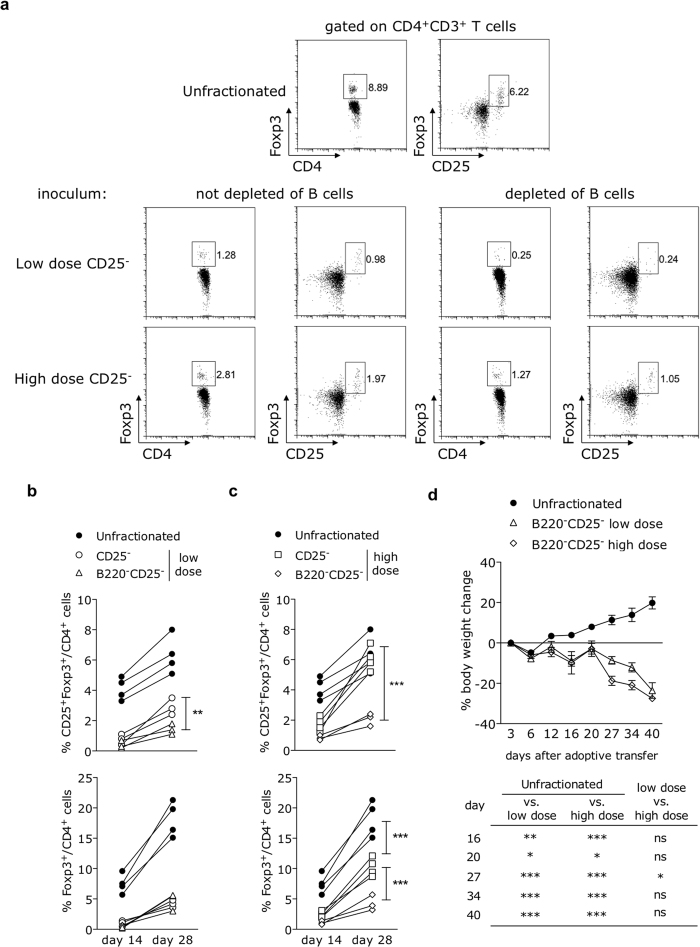
The peripheral emergence of Treg cells is not observed when CD25^+^ cell-depleted T lymphocytes expand in the absence of B cells. B6.*Rag2*^−/−^ mice were adoptively transferred with either unfractionated splenocytes, splenocytes depleted of CD25^+^ cells (20 or 40 × 10^6^ cells per mouse) or splenocytes depleted of both CD25^+^ and B220^+^ cells (10 or 20 × 10^6^ cells per mouse) isolated from adult C57BL/6 donors and the blood frequencies of regulatory T cells were determined by flow cytometry at 14 and 28 days after injection. (**a**) Dot-plots representative of one animal within each experimental group show the percentages of Foxp3^+^ (left) and CD25^+^Foxp3^+^ (right) cells found in total CD4^+^CD3^+^ T cells 14 days after adoptive transfer. Numbers plotted in each quadrant refer to the percentage of events accumulated in CD4^+^ T lymphocyte gate. (**b,c**) Changes in CD25^+^Foxp3^+^ (top) and Foxp3^+^ (bottom) over time are shown within each mouse injected with either (**b**) low or (**c**) high doses of CD25^−^ splenocytes depleted or not of B cells. Data include all experimental animals in each condition, with at least three mice per group (mean ± SEM). (**d**) Mice injected with either the low or high dose of B220^−^CD25^−^ cells were monitored for body weight changes every three days and representative time-points are shown. Insert table (bottom) depicts statistical significance between indicated groups at relevant time-points. *p < 0.05, **p < 0.01, ***p < 0.001; not significant (ns): p > 0.05.

Rag^−/−^ hosts reconstituted with either unfractionated or CD25^−^ splenocytes (both containing approximately 60% of B cells) displayed very low frequencies of circulating B lymphocytes two weeks after transfer (Supplementary Figure 2B). Even though B cells declined *in vivo*, their presence in the inoculum was decisive to the rise of Treg cell frequencies. The early augmentation in the frequencies of overall Foxp3^+^ or CD25^+^Foxp3^+^ T cells, observed 14 days post-transfer, was significantly impaired in the absence of B cells in recipients of either the low or high dose of CD25^−^ splenocytes (Fig. 4a).

The enlargement of the regulatory CD4^+^ T cell subset over time was also dependent on the B cell presence in the inoculum. The lack of B cells did not affect the slight time-dependent elevation of total Foxp3^+^ cells in low dose-injected hosts (Fig. 4b, bottom), and only moderately impaired the rise of CD25-expressing Foxp3^+^ cells (Fig. 4b, top). In remarkable contrast, increases of both total Foxp3^+^ and CD25^+^Foxp3^+^ are markedly inhibited in mice injected with the high dose of B220^−^CD25^−^ splenocytes, although CD25^+^Foxp3^+^ cells were more affected by B cell deprivation (Fig. 4c). Notably, the circulating levels of the CD25^+^Foxp3^+^ subset within CD4^+^ T cells were equivalent between recipients of either unfractionated or high-dose CD25^−^ splenocytes 28 days after transfer (Fig. 4c, top). CD25^+^Foxp3^+^ Treg cells were reported as the subtype stably committed to the regulatory phenotype that displays higher suppressive potential^22^ and, consistent with that, the inability to rescue peripheral CD25^+^Foxp3^+^ Treg numbers in the absence of B cells correlated with development of severe colitis (Fig. 4d). These results unraveled an unappreciated role for B cells as important modulators of T cell-induced colitis, through the generation of CD4^+^CD25^+^Foxp3^+^ Treg cells.

As the increase of Treg cell numbers in lymphopenic recipients was significantly compromised in the absence of B lymphocytes, we investigated if global CD4^+^ T cell homeostasis would also be affected. As expected, the enrichment in peripheral Treg cell frequencies observed in recipients of the high-dose CD25^−^ splenocytes correlated with a significant decrease in the CD44/CD62L ratio when compared to hosts given the low dose of CD25^−^ splenocytes (Supplementary Figure 3A, middle panels). This is in accordance with the reported ability of Treg cells to inhibit lymphopenia-induced proliferation of naïve T cells^23^,^24^. Noteworthy, the regulation of CD44/CD62L ratio was drastically impaired when expansion of CD25^−^ splenocytes took place in the absence of B cells (Supplementary Figure 3A, bottom panels), as summarized in Supplementary Figure 3B. Overall, these findings demonstrate that the B-cell driven augmentation of Treg cells is relevant for the control of the peripheral CD4^+^ T cell homeostasis.

### The initial size of the CD4^+^CD25^−^ T cell pool decisively influences B cell-dependent increase of peripheral Treg cell frequencies

The data obtained from the experiments described above strongly suggested that the absolute number of T cells transferred to the lymphopenic host might be crucial for the decision between disease induction *versus* protection against immunopathology. However, not only T cells, but also B cells, have been augmented in the protective inoculum. To determine whether the B cell-driven augmentation of peripheral Treg cell frequencies relies on a numerical increase of either B or T lymphocyte populations in the inoculum, Rag^−/−^ hosts injected with a given number of CD4^+^CD25^−^ T cells (either 3 or 6 × 10^6^) also received an amount of B cells corresponding to the numbers present either in the colitogenic low dose (10 × 10^6^) or in the colitis-protective high dose (25 × 10^6^) of CD25^−^ splenocytes (Supplementary Figure 4A,B).

**Figure 5:**
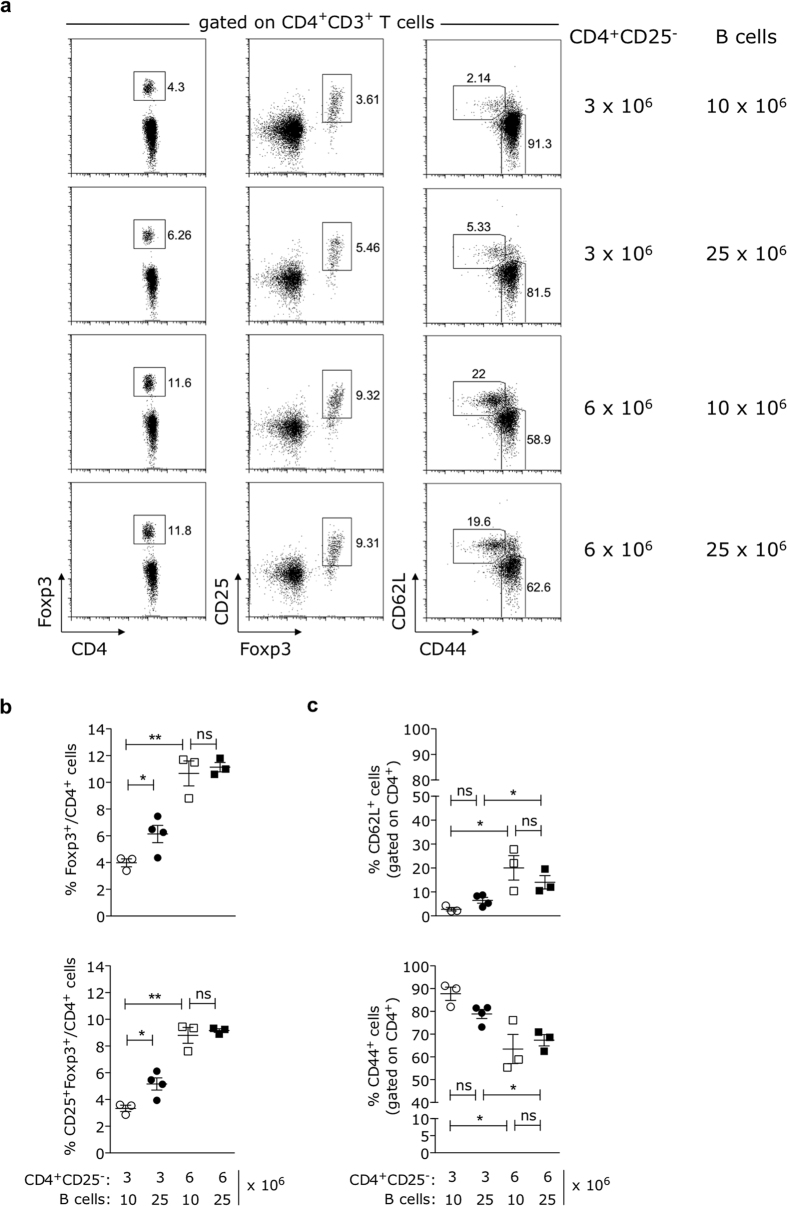
The B cell-mediated increase of Treg cell levels requires an enlarged CD4^+^CD25^−^ T cell pool. B6.*Rag2*^−/−^ mice were adoptively transferred with CD4^+^-enriched splenocytes depleted of CD25^+^ cells (CD4^+^CD25^−^), isolated from adult C57BL/6.Foxp3-GFP donors, along with different numbers of B lymphocytes purified from age- and sex-matched B6 donors. The relative frequencies of Foxp3^+^ Treg cells, as well as effector-like CD44^high^CD62L^−^ and naïve CD44^−^CD62L^high^ T cells, were determined in blood by flow cytometry at day 28 after injection. Absolute numbers of B (10 × 10^6^ and 25 × 10^6^) and T (3 × 10^6^ and 6 × 10^6^) cells used for injection were equivalent to those present in low (20 × 10^6^) and high (40 × 10^6^) doses of CD25^−^ splenocytes and are indicated in the figure. **(a)** Dot-plots representative of one animal within each experimental group show the percentages of Foxp3^+^ (left column), CD25^+^Foxp3^+^ (middle column) and CD44^+^/CD62L^+^ (right column) cells found in total CD4^+^CD3^+^ T lymphocyte gate. **(b,c)** Data including all experimental animals in each condition (with at least three mice per group, mean ± SEM) show blood frequencies of **(b)** Foxp3^+^ (top) and CD25^+^Foxp3^+^ (bottom) and **(c)** CD62L^+^ (top) CD44^+^ (bottom) cells gated as in (**a**). *p < 0.05, **p < 0.01; not significant (ns): p > 0.05.

The injection of a high number of B lymphocytes along with the low number of CD4^+^CD25^−^ T cells only moderately increased Treg cell frequencies (Fig. 5a, middle top row and Fig. 5b) over the values found in animals reconstituted with the low dose of CD25^−^ splenocytes (Fig. 5a, top row and Fig. 5b). In contrast, mice reconstituted with the large number of CD4^+^CD25^−^ T cells showed the highest increase in the peripheral frequencies of Foxp3^+^ Treg cells regardless of the amount of B lymphocytes co-injected (Fig. 5a, middle bottom and bottom rows and Fig. 5b), reaching levels comparable to those observed in recipients of the high dose of CD25^−^ splenocytes (Fig. 2b). The emergence of Foxp3^+^ T cells in the recipients of the high dose of CD4^+^CD25^−^ T cells correlated with a significant reduction in the frequencies of CD44^+^CD4^+^ and a reciprocal augmentation in the percentages of CD62L^+^CD4^+^ T cells when compared to hosts reconstituted with the low dose of CD4^+^CD25^−^ inoculum (Fig. 5a, right column and Fig. 5c). Consistent to what was shown in Supplementary Figure 2A, donor-derived B lymphocytes were found in very low percentages soon after adoptive transfer (Supplementary Figure 4C). These results show that the numerical increase of inoculated T cells, but not of B cells, is the key factor required for the growth of the peripheral Treg cell compartment.

### The presence of CD4^+^CD25^−^Foxp3^+^ T cells is necessary for the suppression of intestinal inflammation

**Figure 6:**
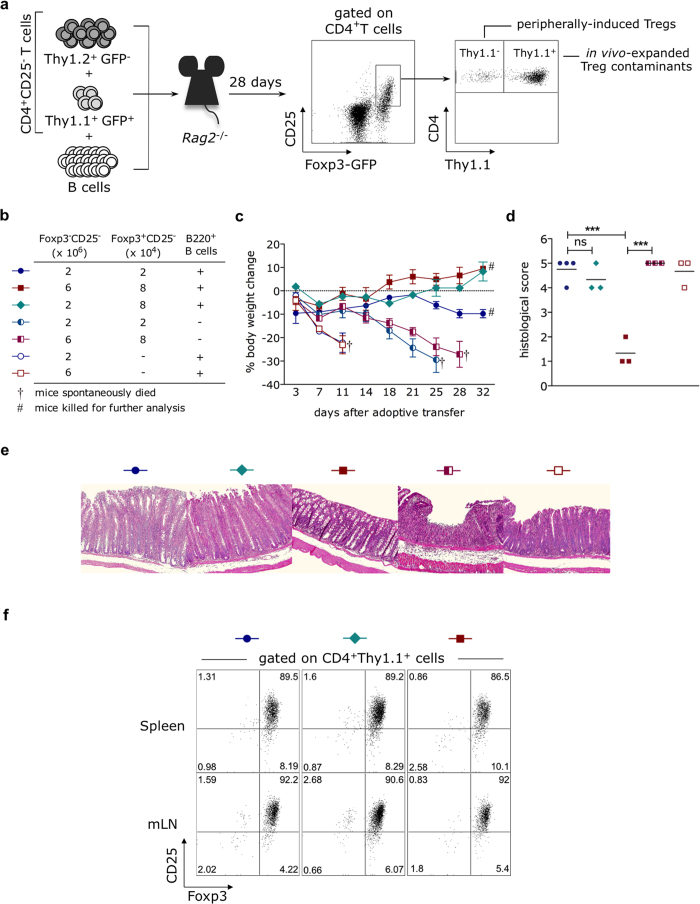
Residual CD25^−^Foxp3^+^ Treg cells are absolutely required for protection against colitis. B6.*Rag2*^−/−^ mice were adoptively transferred with 2 or 6 × 10^6^ FACS-sorted naïve CD4^+^CD25^−^Foxp3^−^ (GFP^−^) T cells, isolated from adult Thy1.2^+^ C57BL/6.Foxp3-GFP donors, in the presence or absence of different numbers (2 or 8 × 10^4^) of co-injected FACS-sorted regulatory CD4^+^CD25^−^Foxp3^+^ (GFP^+^) T cell contaminants (isolated from adult Thy1.1^+^ B6.Ba.Foxp3-GFP mice). Purity of sorted populations were >99.5%. A fixed amount of B lymphocytes (10 × 10^6^) was MACS-purified from age- and sex-matched B6 donors and injected (or not, as a control) along with the T-cell mixture. **(a)** Experimental setup and gating strategies for Treg cell analysis are shown. All cellular analyses were performed 28 days after adoptive transfer. **(b,c)** Mice injected with different T/B-cell mixtures (symbols in the table depict each type of injection) were monitored for body weight change every three days after adoptive transfer. **(d)** H&E-stained colon sections were scored for histopathological signs 28 days after injection. **(e)** Representative photographs of scanned colon sections (horizontal black bars = 200 μm) are shown. Symbols above each photograph refer to the respective groups listed in (**b**). **(f)** Spleens and mesenteric lymph nodes (mLN) were harvested and the expression of Foxp3 and CD25 by CD4^+^Thy1.1^+^ cell contaminants was determined by flow cytometry. Dot-plots are representative of one animal within each experimental group. Numbers plotted in each quadrant refer to the percentage of events accumulated on CD4^+^Thy1.1^+^ gate.

We sought to determine how B lymphocytes support the peripheral augmentation of Treg cell frequencies. Since CD25^−^Foxp3^+^ T cells are not eliminated by magnetic depletion of CD25^+^ cells, B lymphocytes could either promote the proliferation/survival of the minor fraction of CD25^−^Foxp3^+^ T cell contaminants present in the inoculum and/or induce the peripheral conversion of conventional CD25^−^Foxp3^−^ T cells into the CD25^+^Foxp3^+^ regulatory phenotype. To discriminate between these phenomena, *Rag2*^−/−^ mice were adoptively transferred with different populations of CD4^+^CD25^−^ T cells, isolated from congenic Thy-1.1^+^ and Thy-1.2^+^ B6.Foxp3-GFP donors and FACS-sorted according to the expression of the reporter Foxp3-GFP transgene. Foxp3-negative (GFP^−^, Thy1.2^+^) and Foxp3-positive (GFP^+^, Thy1.1^+^) CD4^+^CD25^−^ T cell subsets were mixed at different ratios and injected into lymphopenic recipients along with a fixed amount of purified B cells (Fig. 6a,b). The use of distinct allotypic surface markers allowed us to study the role of B lymphocytes on the survival/expansion of Treg cell contaminants (Thy1.1^+^GFP^+^) and on the generation of pTregs (Thy1.1^−^GFP^+^), as well as the relative importance of each Treg cell subset for the inhibition of colonic inflammation.

Confirming our previous results, mice reconstituted with low numbers of CD25^−^Foxp3^−^ (2 × 10^6^) and CD25^−^Foxp3^+^ (2 × 10^4^) T cells in the presence of B cells displayed severe colonic disease (Fig. 6c–e, blue circle), while their counterparts injected with the higher numbers of the same T-cell subsets (6 × 10^6^ and 8 × 10^4^, respectively) exhibited significant less signs of gut inflammation, as revealed by weight gain (Fig. 6c, red square) and reduced histopathological score (Fig. 6d,e). Corroborating the data shown in Fig. 4d, the protection of colon inflammation required B cell activity, since mice receiving the same T-cell mixtures in the absence of B lymphocytes lost weight over time, eventually coming to death 25–30 days after reconstitution (Fig. 6c–e, half-open symbols).

Importantly, even in the presence of B cells, injections of the low- and high-dose mixtures devoid of CD25^−^Foxp3^+^ regulatory contaminants led to significantly accelerated death of the recipients (Fig. 6c, open symbols), showing that the presence of the small Foxp3^+^ subset among the CD4^+^CD25^−^ population is absolutely required for the control of intestinal inflammation. Only three (out of ten mice) reconstituted with the high dose of conventional T cells in the absence of Treg cell contaminants survived until day 28 post-injection and were included for analysis; not surprisingly, these long-term survivors showed severe colon inflammation (Fig. 6d,e, open square). The Thy1.1^+^CD4^+^Foxp3^+^ cell contaminants, which were CD25^−^ before injection, rapidly gained CD25 expression following *in vivo* expansion, becoming more than 85–90% CD25^+^ (Fig. 6f). Remarkably, CD4^+^Thy1.1^+^ T cell contaminants, which were >99.5% Foxp3^+^ (GFP^+^) at the moment of injection, remained Foxp3^+^ in great majority (Fig. 6f), both in the spleen and in the mesenteric lymph nodes; of note, lack of B cells did not affect the stability of Foxp3 expression among CD4^+^Thy1.1^+^ cells (not shown). The stable expression of Foxp3 within the progeny of Thy1.1^+^ contaminants suggests that this subset kept the regulatory program active, emphasizing their role in the control of mucosal inflammation.

Considering the importance of the CD4^+^CD25^−^Foxp3^+^ T cell subset for the suppression of colitis, we decided to test if the severe colon disease observed in mice receiving the B cell-containing low-dose mixture was due to an insufficient number of regulatory T cell contaminants. For that, we also performed injections of low-dose CD25^−^Foxp3^−^ T cells (2 × 10^6^) along with the same amount of CD25^−^Foxp3^+^ T cells (8 × 10^4^) present in the protective high-dose inoculum. Although gaining weight over time (Fig. 6c, lozenge), these recipients showed severe colonic inflammation, comparable to that observed in hosts reconstituted with the low-dose CD25^−^Foxp3^−^ T cells + low-dose CD25^−^Foxp3^+^ Treg contaminants (Fig. 6d,e). Therefore, suppression of T cell-induced intestinal immunopathology may be achieved with a small number of CD4^+^CD25^−^Foxp3^+^ cells but relies essentially on the initial number of CD4^+^CD25^−^Foxp3^−^ conventional T cell compartment.

### The B cell-mediated increase of pTreg cell numbers is crucial for the suppression of intestinal inflammation

**Figure 7:**
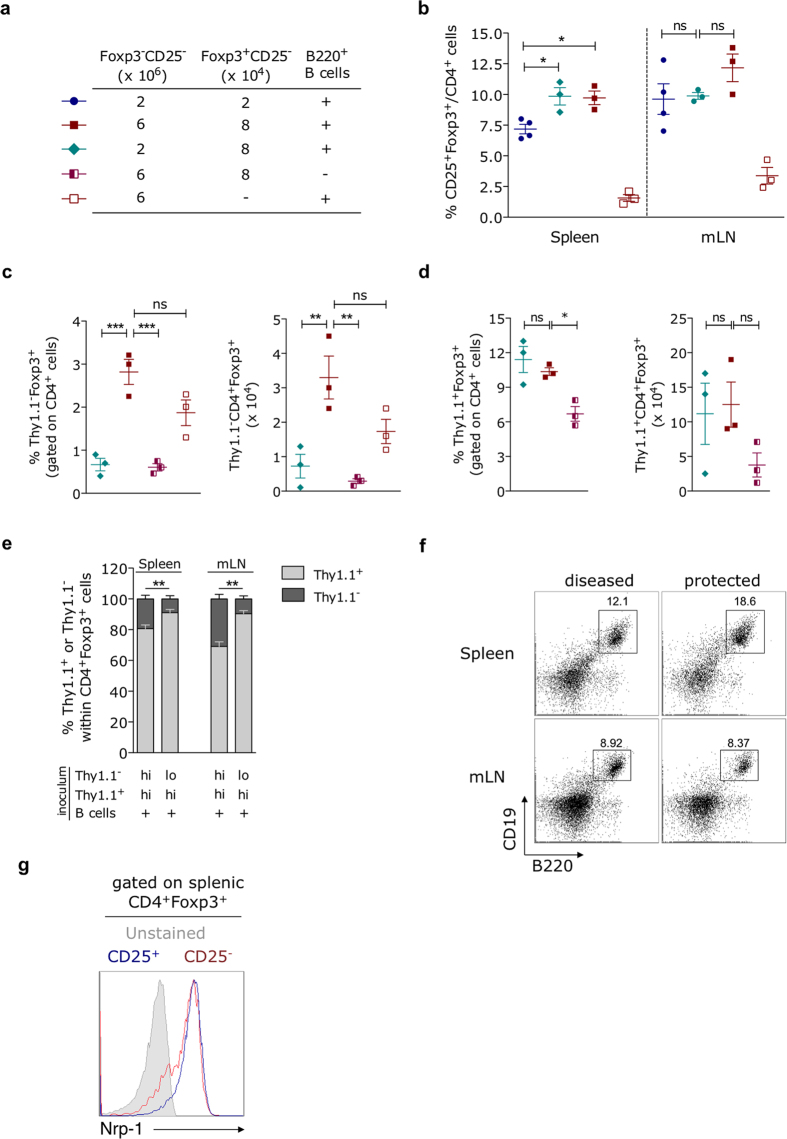
Emergence of pTregs from an enlarged source of naïve T cell precursors is a B cell-dependent process relevant for colitis prevention. Spleens and mesenteric lymph nodes (mLN) were harvested from mice described in Fig. 6 and the frequencies of several lymphocyte populations were determined by flow cytometry. Data including all experimental animals in each condition (with at least three mice individually tested per group, mean ± SEM) are shown. (**a**) Symbols in the table depict each type of injection. (**b**) Frequencies of total CD25^+^Foxp3^+^ Treg cells relative to total CD4^+^ T lymphocytes were estimated in spleen and mesenteric lymph nodes (mLN). (**c,d**) The percentages (left) and absolute numbers (right) of (**c**) peripherally-induced Thy1.1^−^Foxp3^+^ or (**d**) expanded Thy1.1^+^Foxp3^+^ Treg cells relative to the splenic CD4^+^ T cell gate are shown. (**e**) Bar graphs depict the ratio of Thy1.1^−^:Thy1.1^+^ T cells in spleen and mesenteric lymph nodes, relative to the total CD4^+^CD25^+^Foxp3^+^ T cell gate, for protected (red square) and diseased (lozenge) mice. *p < 0.05, **p < 0.01, ***p < 0,001, not significant (ns): p > 0.05. (**f**) Representative flow-cytometric dot-plots show the percentages of CD19^+^B220^+^ B lymphocytes in each lymphoid organ. (**g**) Neuropilin-1 (Nrp-1) surface expression on splenic CD25^−^Foxp3^+^ or CD25^+^Foxp3^+^ CD4^+^ T cells was determined by flow cytometry before injection into lymphopenic recipients. Histogram plot representative of two independent analyses is shown.

The frequencies of total CD4^+^CD25^+^Foxp3^+^ regulatory T cells were analyzed in diseased and protected hosts 28 days after adoptive transfer. Mice reconstituted with the low-dose mixture showed reduced frequencies of CD25^+^Foxp3^+^ T cells in the spleen, when compared to recipients of T-cell mixtures containing the high dose of CD25^−^Foxp3^+^ T cell contaminants (Fig. 7a,b). Surprisingly, diseased mice that received low-dose naïve T cells + high-dose CD25^−^Foxp3^+^ T cell contaminants showed splenic Treg cell frequencies similar to that observed in protected hosts reconstituted with the high-dose mixture (Fig. 7a,b). This result suggests that Treg cells generated in mice reconstituted with low-dose CD25^−^Foxp3^−^ + high-dose CD25^−^Foxp3^+^ T cell contaminants, despite reaching final frequencies equivalent to those present in protected mice, are unable to maintain intestinal homeostasis.

We then compared the frequencies of expanded (Thy1.1^+^) *versus* peripherally-induced (Thy1.1^−^) Foxp3^+^ Treg cells (relative to total CD4^+^ T cells) between diseased and protected recipients harboring equivalent frequencies of total Treg cells. Noteworthy, diseased mice (Fig. 7c, lozenge) showed significantly reduced proportions of splenic peripherally-induced Treg cells when compared to protected counterparts (Fig. 7c, red square). The emergence of pTreg cells was significantly impaired in the absence of B lymphocytes (Fig. 7c, half-open square). Remarkably, the numbers of Thy1.1^+^ contaminant-derived Treg cells were similar between protected and diseased hosts and were not significantly impacted by B-cell deprivation (Fig. 7d). Although no significant differences in the frequencies of total Treg cells were observed between protected and diseased hosts in the mesenteric lymph nodes (Fig. 7b), this organ harbored significantly higher proportions of pTreg cells in protected mice (Fig. 7e). Importantly, the defective accumulation of pTreg cells in diseased hosts does not seem to be due to insufficient B cell numbers, as they show similar frequencies of residual CD19^+^B220^+^ lymphocytes when compared to protected mice, in both spleen and mesenteric lymph nodes (Fig. 7f). Overall, these results strongly suggested that the B cell-dependent generation of pTreg cells, as a consequence of a numerically enlarged naïve T cell compartment, is absolutely required for the protection against intestinal inflammation.

In summary, we demonstrated that the selective appearance of CD4^+^CD25^+^Foxp3^+^ pTreg cells in recipients of the high-dose inoculum of Treg-depleted CD4^+^ T cells is a B cell-dependent process linked to the prevention of large intestine inflammation. Our results favor a model whereby the proper suppression of colitis induced by depletion of Treg cells is only reached when three conditions are fulfilled: (i) numerically widening of CD4^+^CD25^−^Foxp3^−^ naïve T cell source; (ii) B cell-licensed peripheral generation of CD4^+^CD25^+^Foxp3^+^ pTregs and (iii) presence of residual CD4^+^CD25^−^Foxp3^+^ thymic-derived Treg cells.

## Discussion

In the present work, using a model of T cell-induced colitis, we found that the amount of potentially colitogenic cells injected into Rag^−/−^ mice is decisive to the outcome of the disease: animals injected with a high number of CD25^−^ splenocytes were protected against colon inflammation. This unexpected result initially seemed paradoxical, as the absolute numbers of potentially colitogenic CD4^+^ T cells are two-fold higher in the inoculum of 40 × 10^6^ CD25^−^ splenocytes, which could presumably exacerbate and/or accelerate disease induction. However, after expansion in lymphopenic hosts, such dose did not give rise to augmented T cell frequencies when compared to the inoculum of 20 × 10^6^ CD25^−^ splenocytes and, in fact, T cells reached levels closer to those seen in recipients of unfractionated splenocytes. Importantly, protection from colitis, confirmed macroscopically and histologically, was consistent with a significant and stable elevation in the peripheral frequencies of regulatory CD4^+^Foxp3^+^ T cells, corroborating several studies showing that regulatory T cells are the major immune cell type mediating colitis suppression^5^.

Lymphopenia-induced proliferation of a high number of enriched CD4^+^CD25^−^ T cells, an inoculum depleted of the major non-T cell populations as well as CD8^+^ and gamma-delta T cells, gave rise to colon disease as severe as that observed in hosts receiving the low number of CD4^+^CD25^−^ T cells. This is consistent with data previously reported^17^, confirming that augmented numbers of purified naïve CD4^+^ T cells adoptively transferred to T/B cell-deficient hosts do not inhibit colitis development. However, experiments published so far had never assessed whether the same outcome would happen if conventional T cells were transferred along with other cell types not present in the lymphopenic recipients and allowed to expand in their presence.

B lymphocytes may be decisive for the establishment of normal Treg cell numbers under steady-state^19^,^20^ and pathologic conditions^25^,^26^. We showed here that this cell type is absolutely required to allow the selective emergence of Treg cells in mice injected with the high dose of CD25^−^ splenocytes. Rag^−/−^ mice are able to generate dendritic cells competent to mediate lymphopenia-induced proliferation of CD4^+^ T cells under steady-state conditions^27^, as well as T cell-mediated inflammatory or regulatory responses^28^,^29^,^30^. However, even in the presence of competent DCs, B cells were required for the recovery of Treg cell frequencies under lymphopenia, which places this lymphoid cell type as the crucial mediator of the enlargement of Treg cell pool *in vivo*.

Adoptive transfer of mature B lymphocytes results in poor B cell recovery from peripheral lymphoid organs soon after reconstitution^31^. Nonetheless, detectable levels of CD19^+^B220^+^ B lymphocytes were found in the blood and peripheral lymphoid organs of our hosts more than one month after adoptive transfer of B cell-containing lymphoid populations. Although we have not directly assessed whether the long-term presence of B lymphocytes is required to maintain peripheral Treg cell numbers, it is likely that the permanent interaction of T cells with a reminiscent self-renewing or long-lived B cell population^32^ may underlie the time-dependent rise in Treg cell frequencies. Alternatively, a time-delimited interplay between B and T cells, sufficient to produce long-term effects in the CD4^+^ T cell compartment that propitiate the recovery of regulatory T cell levels following lymphopenic reconstitution, remains a possibility.

The strategy used here to address the importance of B cells to the Treg cell homeostasis, consisting of the adoptive transfer of purified B lymphocytes to Rag^−/−^ recipients, could raise concerns regarding the risk of contamination by minor populations. The use of mice congenitally deficient for B lymphocytes as donors of colitogenic T cells would, therefore, be a straightforward approach to confirm the pro-tolerogenic role of B lymphocytes described in our model. However, several studies have suggested a considerable influence of B cells in shaping the T cell repertoire^33^,^34^,^35^,^36^,^37^,^38^. As the central question of the present manuscript was to investigate the impact of the size of Tconv population on the peripheral T cell homeostasis, the adoptive transfer of an altered T cell repertoire could bias our results. To avoid this undesirable interference, the best experimental approach was to combine purified populations of wild-type T and B lymphocytes in order to exploit the role of the latter on the emergence of peripheral Treg cells. Detailed analyses of the inocula used in all experiments revealed a level of purity extremely high, which allowed us to reliably support the conclusions described throughout the manuscript.

The contribution of B lymphocytes to the pathogenesis of T cell-mediated autoimmunity is controversial, with evidences pointing to protective and pathogenic roles. In the context of experimental autoimmune encephalomyelitis (EAE), it has been shown that B-cell depletion therapy ameliorates central nervous system (CNS) inflammation through ablation of pathogenic IL-6-producing B lymphocytes^39^. Accordingly, B cell-restricted IL-6 deficiency impaired Th17 cell response and reduced susceptibility to EAE^40^. It has also been suggested that lack of B cells may favor Treg cell suppressive activity against spontaneous autoimmune thyroiditis^41^.

On the other hand, a number of studies have proposed that B cells are endowed with regulatory properties, being required to suppress T cell-mediated autoimmune diseases. B cell-deficient mice are more susceptible than wild-type controls to antigen-induced EAE^42^,^43^ and B cell-mediated suppression of autoimmunity may be achieved by IL-10-independent^44^ or -dependent^45^ mechanisms. Spontaneous colitis in IL-10^−/−^ mice is exacerbated by B cell depletion^44^ and can be attenuated by injection of IL-10-sufficient B cells isolated from the peritoneal cavity^46^.

Peritoneal cavity-derived B cells, although naturally enriched for IL-10-producing cells, were solely able to ameliorate, but not to prevent, T cell-induced colon inflammation when transferred to Rag^−/−^ recipients along with colitogenic CD4^+^CD25^−^CD45RB^hi^ T cells^46^. Additionally, it has been shown that B lymphocytes gain the ability to inhibit T cell-induced colitis, in an IL-10-dependent fashion, only after being exposed to enterobacteria *ex vivo*^47^, which is consistent with data showing that TLR-activated B cells are more potent suppressors of EAE^48^. We showed that the B cell-dependent protection observed in our model does not require pre-treatment of B lymphocytes with enteroantigens, TLR agonists or artificial enrichment in IL-10-producing cells. The results presented here, therefore, unraveled a previously unappreciated ability of splenic B cells to mediate prevention of T cell-induced colitis, instead of only amelioration, through enlargement of Treg cell numbers. The molecular aspects underlying the pro-tolerogenic properties of B lymphocytes in our model need further investigation, specially concerning the role of cognate interactions and/or the secretion of immunoregulatory cytokines.

In addition to the major presence of B lymphocytes, the careful analysis of our CD25^−^ cell inoculum revealed a very low percentage of Treg cell contaminants, mostly corresponding to CD4^+^CD25^−^Foxp3^+^ cells that cannot be excluded by the magnetic depletion of CD25^+^ cells. It has been proposed^49^ that this subset originates in the thymus as CD25^+^Foxp3^+^ Treg cells, but loses CD25 expression in the periphery as a result of attenuation or absence of activating signals, albeit CD25 expression can be regained under certain circumstances, such as lymphopenic settings. In line with this view, we showed that the majority of splenic CD4^+^CD25^−^Foxp3^+^ T cells express Nrp-1 (Fig. 7g), a surface marker predominantly expressed by thymic-derived Treg cells in steady-state conditions^50^,^51^. Although both low- and high- dose-injected mice received T cells harboring the same frequencies of Foxp3^+^ contaminants, only reconstitution with higher absolute numbers of CD25^−^ splenocytes resulted in elevation of Treg cell frequencies to levels up to 13%. Therefore, the intriguing question is why regulatory T cells were uniquely enriched when the number of potentially colitogenic cells was also increased.

The adoptive transfer of highly-purified T cell populations into Rag^−/−^ recipients helped us to unravel the critical role of CD25^−^Foxp3^+^ T cell contaminants on the restoration of peripheral Treg cell numbers and disease prevention. Although present in very low frequencies within the CD25^−^ splenocyte inoculum, this cell subset was able to rapidly expand under lymphopenic settings and dominate the Treg cell pool. Consistent to published data^49^ and confirmed in our model, this population rapidly regained CD25 expression after lymphopenia-induced proliferation, probably as a consequence of competitive advantage provided by their self-reactive TCR repertoire. In the absence of these Treg cell population, the mucosal inflammation became completely uncontrolled and mice died quickly after adoptive cell transfer.

Importantly, the CD25^−^Foxp3^+^ T cells have been referred to as unstable Treg cells, which are not committed to the regulatory phenotype^22^ and may lose Foxp3 expression, becoming pathogenic^52^,^53^ under certain conditions. However, in our model, Foxp3 expression by this population was quite stable, with more than 96% of total CD4^+^Thy1.1^+^ remaining Foxp3^+^ after peripheral expansion. This observation suggests that these cells are playing suppressive roles, rather than pathogenic ones, over the induction of mucosal inflammation. It is important to emphasize that this population, comprising a minor fraction of the Treg cell compartment from an young adult mouse, seems to be endowed with the intrinsic ability of rapid expansion and is able to retain its suppressive functions even in the absence of the stably committed CD25^+^Foxp3^+^ Treg cells, which were depleted from the inoculum and never colonized the lymphopenic hosts in our model. This conclusion is supported by our data showing that the majority of CD25^+^Foxp3^+^ T cells found in recipients 28 days after reconstitution express Thy1.1 and are, therefore, derived from the contaminant CD25^−^Foxp3^+^ T cell source. In addition, our results suggest that this subset is regulated in a B cell-independent fashion, since neither its number nor the stability of Foxp3 expression was significantly impacted by the lack of B cells.

Although fundamental for colitis prevention, the expansion of CD25^−^Foxp3^+^ Treg cell subset was similar between diseased and protected hosts reconstituted with the same number of CD25^−^Foxp3^+^ Treg cells, indicating that their number is not the essential factor deciding disease *versus* protection. The pTreg cell compartment, instead, was the central element to be increased for achieving protection. The direct role of B lymphocytes on the peripheral conversion of Foxp3^−^ precursors to the regulatory Foxp3^+^ phenotype has been addressed by few studies. Evidences accumulated from them have suggested that B lymphocytes are efficient in promoting peripheral Treg cell generation, by mechanisms that involve CD40-CD40L interactions^54^ and antigen presentation^55^. However, neither of these studies was able to discriminate whether the B-cell-dependent enlargement of peripheral Treg cell compartment was a consequence of tTreg expansion or pTreg formation in secondary lymphoid organs, which raises concerns regarding the exact impact of B cells on the peripheral immunorregulation mediated by CD4^+^Foxp3^+^ Tregs.

By the use of sorted T cell populations bearing different allotypic markers, we could reliably track the fate of CD25^−^Foxp3^−^ (Thy1.1^−^) naïve and CD25^−^Foxp3^+^ (Thy1.1^+^) T cells *in vivo* in the presence or absence of B lymphocytes. The numbers of Thy1.1^−^ pTreg cells, but not of Thy1.2^+^ tTreg cell populations, were greatly reduced in the absence of a functional B cell compartment, which demonstrates that B lymphocytes are selectively involved in pTreg cell generation/establishment, acting as either direct inducers of the peripheral conversion from Foxp3^−^ precursors and/or preferential cellular niches that may mediate the survival/persistence of already peripherally-induced Treg cells. Our work, therefore, points to an absolute requirement of B lymphocytes for the pTreg generation *in vivo*, a concept never raised and/or experimentally supported before. Such a conclusion would not be easily predictable, given that dendritic cells, classically associated with pTreg cell formation, are not numerically or functionally defective in Rag knock-out mice. The data described here also suggest that a minimal threshold of pTreg cells, not only of tTreg cells, must be achieved to afford mucosal homeostasis.

Recently, we have compared the *in vivo* proliferative potential of CFSE-labeled Treg and Tconv populations after adoptive transfer into B-cell-sufficient or -deficient hosts^56^. Interestingly, Tconv cell proliferation is strongly refrained in the presence of B lymphocytes, while Treg cell expansion remained unaffected even under B cell deprivation. This finding suggests that B-cell-mediated inhibition of Tconv cell proliferation contributes to the establishment of peripheral T cell homeostasis. This mechanism may act to prevent exaggerated effector T cell formation in the context of mucosal homeostasis and, additionally, be required for the promotion of pTreg. However, whether suppression of Tconv cell proliferation by B lymphocytes is a pre-requisite for pTreg cell formation is yet to be determined.

Although required for the establishment of mucosal homeostasis, B lymphocytes are incapable of affording prevention of intestinal inflammation when Tconv and residual CD25^−^Foxp3^+^ cells are not present in minimal numbers. By reconstituting lymphopenic recipients with a fixed number of CD25^−^Foxp3^+^ Treg cell contaminants in combination with different numbers of conventional T cells, we confirmed that the augmentation of the latter was the critical requirement to achieve disease prevention. A very constrained conventional CD4^+^ T cell source gave rise to low numbers of pTreg that could not afford protection, even though the levels of expanded Foxp3^+^ tTreg cells were equivalent to those found in the recipients of the high dose of CD25^−^ Tconv cells. In other words, the B-cell dependent augmentation of pTreg cell numbers, here shown to be essential for mucosal health, is almost completely abolished when the Tconv cell pool is narrowed. Our findings provide, therefore, the first demonstration that the size of the CD4^+^CD25^−^Foxp3^−^ Tconv cell compartment, which is the direct source for pTreg cells, is crucial for the regulation of mucosal homeostasis.

It is well established that the activated/effector and regulatory CD4^+^ T cell numbers are indexed *in vivo* and kept stable under steady-state through a mechanism that involves signaling by interleukin-2 (IL-2) via CD25, the high-affinity IL-2Rα chain expressed constitutively by most CD4^+^Foxp3^+^ T cells^57^,^58^. According to that paradigm, an imbalanced ratio Treg/Teff present in the peripheral compartment would be restored to a normal physiological proportion regardless of the initial number of Treg cells. Therefore, in both low- and high-dose injected hosts, the IL-2 produced by conventional T cells during lymphopenia-induced proliferation is expected to be efficiently sequestered by either the minor fraction of contaminants CD25^+^Foxp3^+^ or CD25^−^Foxp3^+^ cells that had upregulated CD25 expression after proliferation, leading to the expansion the regulatory T cell numbers to a normal amount, likely sufficient to control colitis. However, this was found not to be the case in our experiments, where the expanded numbers of Treg cells, required to maintain intestinal homeostasis, were observed only in the high-dose injected hosts. It is possible, therefore, that the quantitative differences that are put forward to explain protection *versus* disease in our model (for example, augmentation in total Treg cell numbers and selective enrichment of pTreg in the mesenteric lymph nodes of protected mice) may be better interpreted in the light of qualitative changes related to diversification of the T cell repertoire.

Recent reports have shed a light on the importance of repertoire diversification for the regulation of autoimmune and inflammatory reactions and also for the peripheral persistence of CD4^+^ T cell clones. *In vivo*-generated pTreg cells seem to be essential for the control of colitis and autoimmune activation^11^,^12^, collaborating for immune regulation by adding new specificities (derived from conventional T cells) to the established repertoire of tTreg cells. More recently, it has been reported that the repertoires of conventional T and tTreg cells specific for the same antigen are different^59^. Specificities originated in the conventional T cell pool can, thus, be crucial for suppression of immune responses if those clonotypes peripherally acquire Foxp3 expression. We believe that the enrichment of diversity within the conventional T cell fraction could allow for the appearance of clonotypes reactive to peptides relevant for colitis induction, either absent or present in insufficient numbers in the low-dose CD25^−^ splenocyte inoculum. After lymphopenia-induced proliferation, those conventional clonotypes with appropriate antigen specificity could undergo peripheral conversion to regulatory phenotype and now occupy previously empty niches, reflecting on the numerical augmentation seen in the colitis-free hosts given the high dose of CD25^−^ splenocytes. In support to this idea, the importance of a highly diversified T cell repertoire for restraining pathogenic monoclonal-driven responses and controlling CD4^+^ T cell numbers was demonstrated^60^.

Recent studies also suggested that a reduced diversity of Treg cell repertoire^61^ and the lack of B lymphocytes^62^ negatively impact the regulation of intestinal homeostasis, independently corroborating our work in important points. The data presented here, however, focus on aspects not appreciated by the other two reports, contributing to unravel a fundamental cellular network required for the control of T-cell-mediated colitis. Our findings show that, in a condition where the peripheral number of tTreg has been severely diminished, the prevention of colitis requires the concerted action of three central elements: B lymphocytes, an enlarged pool of conventional CD4^+^CD25^−^Foxp3^−^ T cells (the source of pTreg) and residual CD4^+^CD25^−^Foxp3^+^ tTregs. We propose a model where the action of residual CD4^+^CD25^−^Foxp3^+^ cells are indispensable to suppress the initial activation/proliferation of Tconv cells in response to environmental antigens, while B lymphocytes act to promote/maintain the peripheral conversion of pTreg cells, which reaches a level adequate to maintain mucosal homeostasis only when the Tconv cell source is wide enough.

In conclusion, our results suggest that inhibition of the undesirable effects of T cell-mediated immune responses may not be successfully achieved by restraining the overall number of potentially pathogenic T cells. This particular aspect may be relevant for autoimmunity. The clinical treatment of autoimmune diseases often relies on therapeutic strategies that dampen global T cell function and/or numbers. Although initially beneficial for the control of T cell-mediated immunopathology, prolonged T cell-targeted therapies may lead not only to immunodeficiency, rendering patients susceptible to opportunistic infections, but also to disruption of immune regulation. The data presented here support the notion that the arbitrary reduction of overall T cell numbers, typical of immunosuppressive drug-based therapy, may be unsuccessful to establish dominant tolerance, since it can be accompanied by a non-estimated contraction of T cell diversity. The maintenance of a high-degree TCR diversity may be especially important to reach the adequate level of B cell-dependent conversion to and/or maintenance of peripherally-induced regulatory T lymphocytes, functionally able to block disease induction and/or progression in collaboration with thymic-derived regulatory counterparts.

## Methods

### Mice

Six-to-eight week-old B6.SJL-*Ptprc*^*a*^ *Pep3*^*b*^/BoyJ (CD45.1^+^ B6.SJL), C57BL/6, C57BL6.Foxp3-GFP or B6.Ba/Foxp3-GFP mice were used as donors and B/T cell-deficient *Rag2*^−/−^ mice (on C57BL/6 background) were used as hosts of spleen cell suspensions. All animals were bred under specific pathogen-free conditions at NAL/Universidade Federal Fluminense, Niterói/RJ, Brazil. The experimental protocols used in this work were approved by The Ethics Committee for Animal Experimentation (Comissão de Ética no Uso de Animais, CEUA) of the Universidade Federal Fluminense (approval register #512). Animals received humane care and all the protocols were performed in compliance with the approved guidelines prioritized by the “Principles of Laboratory Animal Care” (National Society for Medical Research, USA) and the National Council for Controlling Animal Experimentation (CONCEA/MCTI, Brazil).

### Cell purifications and adoptive cell transfers

Sterile single-cell suspensions, obtained through mechanical disruption of spleens, were diluted in phosphate-buffered saline (PBS) supplemented with 3% fetal calf serum (FCS). After red cell lysis by ammonium chloride, cells were counted in the presence of trypan blue, ressuspended in PBS supplemented with 0.5% bovine serum albumin (BSA) and 2 mM EDTA (PBS-BSA-EDTA) and incubated with biotin-conjugated monoclonal antibodies for 20 minutes at 4 °C. For CD25^+^ and/or B220^+^ cell depletion, spleen cell suspensions were incubated with anti-CD25 (clone PC61, eBioscience) and/or anti-B220 (Biolegend); for CD4^+^ T cell or B220^+^ B cell enrichment, the antibodies used were anti-B220 (RA3–6B2) or anti-CD4^+^ (GK1.5), respectively, in addition to -CD8α (53–6.7), -γδ (GL3), -CD11b (M1/70), -CD11c (N418) and -CD49d (DX-5), all purchased from Biolegend. Biotin-labeled cells were then incubated with Streptavidin-Beads (Myltenyi) for 15 minutes at 4 °C and washed twice before being loaded onto magnetic collumns. Cell depletions were performed using MACS LD collums (Miltenyi Biotec) and purity of the flow-through fractions were checked by FACS staining before injection. CD25^+^ cell depletion was >95% pure, B220^+^ cell depletion was >99% pure and CD4^+^ T cell enrichment was 87.5–92% pure. For cell sorting, splenic cell suspensions, isolated from Thy1.1^+^ B6.Ba/Foxp3-GFP and Thy1.2^+^ C57BL6.Foxp3-GFP mice, were enriched for CD4^+^ T cells by MACS-based negative selection and then incubated with APC-conjugated anti-CD4 (GK1.5, Biolegend) and PE-conjugated anti-CD25 (PC61, eBioscience) in PBS-BSA-EDTA for 20 min. After washing, CD25^−^Foxp3.GFP^+^ (Thy1.1^+^) regulatory T cells and CD25^−^Foxp3.GFP^−^ (Thy1.2^+^) conventional T cells were purified using a MoFlo flow cytometer (DakoCytomation). The purity of sorted populations was >99%. Unfractionated splenocytes, MACS-purified or FACS-sorted fractions were counted, ressuspended in sterile PBS and injected intravenously, via retro-orbital plexus, into adult *Rag2*^−/−^ mice.

### FACS Staining

Immunofluorescence staining of peripheral blood, spleen and lymph node single-cell suspensions was performed using the following monoclonal antibodies: allophycociannin (APC)-CD4 (GK1.5) and phycoerythrin (PE) anti-CD25 (PC61.5), purchased from eBioscience, and fluorescein isothiocyanate (FITC)-anti-CD8 (YTS 156.7.7) and peridinin chlorophyll protein (PerCP)-anti-CD45.1 (A20), purchased from BioLegend. For neuropilin-1 staining, PE anti-Nrp-1/CD304 (3E12, Biolegend) was used in conjunction with APC anti-CD25 (PC61.5, eBioscience). Cell membrane permeabilization and Foxp3 intracellular staining, using either Alexa488- or biotin-conjugated anti-Foxp3 monoclonal antibody (FJK-16 s, eBioscience) in conjunction with PECy7-streptavidin, were performed according to the instructions contained in eBioscience commercial kit. Stained cells were acquired with AccuriC6 (AccuriCytometers) flow cytometer and analyzed with FlowJo v8.7 software (Treestar).

### Colitis induction and examination

*Rag2*^−/−^ mice were adoptively transferred with either 20 × 10^6^ or 40 × 10^6^ CD25^+^ cell-depleted splenocytes and weighed every 2–3 days after injection. Additionally, mice were periodically monitored for signs of diarrhea and euthanized 28 days post-injection for macroscopic and histological analysis of colons.

### Histologic preparation and determination of histopathological score

*Rag2*^−/−^ recipients were euthanized four weeks after adoptive transfer and the excised colons were fixed overnight in formalin-buffered Millonig solution (pH 7.2), processed and embedded in paraplast (Sigma, St Louis, Missouri). Five μm thick sections were stained with haematoxylin-eosin (HE) to assess morphological alterations (such as crypt dilatation, erosion of mucosal layer, frequency of mucin-secreting globet cells) and cellular infiltration. Colon sections were scored according to parameters described elsewhere^63^. Images of cross-sections from at least three experimental and control mice were acquired with either a microdigital camera mounted on a Zeiss Axioplan microscope (Zeiss, Oberkochen, Germany) or a Aperio slide scanner (Leica Biosystems) integrated with the software Aperio ImageScope.

### Statistical analysis

GraphPad Prism Software (version 5.0) was used to perform all the analyses and graphs shown. Data from analyzes of cell frequencies or histopathological scores were compared among groups using one-way ANOVA test with Turkey post-test. Curves depicting body weight changes were analyzed using two-way ANOVA with Bonferroni post-test. Differences between groups were considered statistically significant when p values were below 0.05 and not significant when p values were above 0.05 (ns). Means ± SEM are depicted in the figures.

## Additional Information

**How to cite this article**: Canto, F.B. *et al*. Enlarged colitogenic T cell population paradoxically supports colitis prevention through the B-lymphocyte-dependent peripheral generation of CD4^+^Foxp3^+^ Treg cells. *Sci. Rep*. **6**, 28573; doi: 10.1038/srep28573 (2016).

## Supplementary Material

Supplementary Information

## References

[b1] Uhlig H. H. & Powrie F. Mouse models of intestinal inflammation as tools to understand the pathogenesis of inflammatory bowel disease. Eur. J. Immunol. 39, 2021–2026 (2009). 19672896 10.1002/eji.200939602

[b2] Fowell D., McKnight A. J., Powrie F., Dyke R. & Mason D. Subsets of CD4^+^ T cells and their roles in the induction and prevention of autoimmunity. Immunol. Rev. 123, 37–64 (1991). 1684782 10.1111/j.1600-065x.1991.tb00605.x

[b3] Powrie F., Leach M. W., Mauze S., Caddle L. B. & Coffman R. L. Phenotipically distinct subsets of CD4^+^ T cells induce or protect from chronic intestinal inflammation in C.B-17 *scid* mice. Int. Immunol. 5, 1461–1471 (1993). 7903159 10.1093/intimm/5.11.1461

[b4] Uhlig H. H. . Characterization of Foxp3^+^CD4^+^CD25^+^ and IL-10-secreting CD4^+^CD25^+^ T cells during cure of colitis. J. Immunol. 177, 5852–5860 (2006). 17056509 10.4049/jimmunol.177.9.5852 PMC6108413

[b5] Mottet C., Uhlig H. H. & Powrie F. Cutting Edge: Cure of colitis by CD4^+^CD25^+^ regulatory T cells. J. Immunol. 170, 3939–3943 (2003). 12682220 10.4049/jimmunol.170.8.3939

[b6] Bailey-Bucktrout S. L. & Bluestone J. A. Regulatory T cells: stability revisited. Trend. Immunol. 32, 301–306 (2011). 10.1016/j.it.2011.04.002 PMC3129467 21620768

[b7] Jordan M. S. . Thymic selection of CD4^+^CD25^+^ regulatory T cells induced by an agonist self-peptide. Nat. Immunol. 2, 301–306 (2001). 11276200 10.1038/86302

[b8] Apostolou I., Sarukhan A., Klein L. & von Boehmer H. Origin of regulatory T cells with known specificity for antigen. Nat. Immunol. 3, 756–763 (2002). 12089509 10.1038/ni816

[b9] Lee H. M., Bautista J. L., Scoot-Browne J., Mohan J. F. & Hsieh C. S. A broad range of self-reactivity drives thymic regulatory T cell selection to limit responses to self. Immunity 37, 475–486 (2012). 22921379 10.1016/j.immuni.2012.07.009 PMC3456990

[b10] Bilate A. M. & Lafaille J. J. Induced CD4^+^Foxp3^+^ regulatory T cells in immune tolerance. Annu. Rev. Immunol. 30, 733–758 (2012). 22224762 10.1146/annurev-immunol-020711-075043

[b11] Haribhai D. . A central role for induced regulatory T cells in tolerance induction in experimental colitis. J. Immunol. 182, 3461–3468 (2009). 19265124 10.4049/jimmunol.0802535 PMC2763205

[b12] Haribhai D. . A requisite role for induced regulatory T cells in tolerance based on expanding antigen receptor diversity. Immunity 35, 109–122 (2011). 21723159 10.1016/j.immuni.2011.03.029 PMC3295638

[b13] Bilate A. M. & Lafaille J. J. It takes two to tango. Immunity 35, 6–8 (2011). 21777793 10.1016/j.immuni.2011.07.003

[b14] Gad M. Regulatory T cells in experimental colitis. Curr. Top. Microbiol. Immunol. 293, 179–208 (2005). 15981481 10.1007/3-540-27702-1_9

[b15] Francisco L. M. . PD-L1 regulates the development, maintenance and function of induced regulatory T cells. J. Exp. Med. 206, 3015–3029 (2009). 20008522 10.1084/jem.20090847 PMC2806460

[b16] Lagranderie M. . *Mycobacterium bovis* Bacillus Calmette-Guérin killed by extended freeze-drying reduces colitis in mice. Gastroenterology 141, 642–652 (2011). 21683076 10.1053/j.gastro.2011.05.002

[b17] Coombes J. L., Robinson N. J., Maloy K. J., Uhlig H. H. & Powrie F. Regulatory T cells and intestinal homeostasis. Immunol. Rev. 204, 184–194 (2005). 15790359 10.1111/j.0105-2896.2005.00250.x

[b18] Darrasse-Jèze G. . Feedback control of regulatory T cell homeostasis by dendritic cells *in vivo*. J. Exp. Med. 206, 1853–1862 (2009). 19667061 10.1084/jem.20090746 PMC2737156

[b19] Sun J., Flach C., Czerkinsky C. & Holmgren J. B lymphocytes promote expansion of regulatory T cells in oral tolerance: powerful induction by antigen coupled to cholera toxin B subunit. J. Immunol. 181, 8278–8287 (2008). 19050244 10.4049/jimmunol.181.12.8278

[b20] Shah S. & Qiao L. Resting B cells expand a CD4^+^CD25^+^Foxp3^+^ Treg population via TGF-β3. Eur. J. Immunol. 38, 2488–2498 (2008). 18792402 10.1002/eji.200838201

[b21] Canto F. B. . Susceptibility of neonatal T cells and adult thymocytes to peripheral tolerance to allogeneic stimuli. Immunology 125, 387–396 (2008). 18462348 10.1111/j.1365-2567.2008.02855.x PMC2669142

[b22] Komatsu N. . Heterogeneity of natural Foxp3^+^ T cells: a committed regulatory T-cell lineage and an uncommitted minor population retaining plasticity. Proc. Natl. Acad. Sci. USA 106, 1903–1908 (2009). 19174509 10.1073/pnas.0811556106 PMC2644136

[b23] Min B., Yamane H., Hu-Li J. & Paul W. E. Spontaneous and homeostatic proliferation of CD4 T cells are regulated by different mechanisms. J. Immunol. 174, 6039–6044 (2005). 15879097 10.4049/jimmunol.174.10.6039

[b24] Winstead C. J., Fraser J. M. & Khoruts A. Regulatory CD4^+^CD25^+^Foxp3^+^ T cells selectively inhibit the spontaneous form of lymphopenia-induced proliferation of naïve T cells. J. Immunol. 180, 7305–7317 (2008). 18490730 10.4049/jimmunol.180.11.7305

[b25] Yanaba K. . A regulatory B cell subset with a unique CD1^hi^CD5^+^ phenotype controls T cell-dependent inflammatory responses. Immunity 28, 639–650 (2008). 18482568 10.1016/j.immuni.2008.03.017

[b26] Watanabe R. . Regulatory B cells (B10 cells) have a suppressive role in murine lupus: CD19 and B10 cell deficiency exacerbates systemic autoimmunity. J. Immunol. 84, 4801–4809 (2010). 20368271 10.4049/jimmunol.0902385 PMC3734559

[b27] Saini M., Pearson C. & Seddon B. Regulation of T cell-dendritic cell interactions by IL-7 governs T-cell activation and homeostasis. Blood 113, 5793–5800 (2009). 19357399 10.1182/blood-2008-12-192252 PMC2700319

[b28] Malmström V. . CD134L expression on dendritic cells in the mesenteric lymph nodes drives colitis in T cell-restored SCID mice. J. Immunol. 166, 6972–6981 (2001). 11359859 10.4049/jimmunol.166.11.6972

[b29] Coombes J. L. & Powrie F. Dendritic cell in intestinal immune regulation. Nat. Rev. Immunol. 8, 435–446 (2008). 18500229 10.1038/nri2335 PMC2674208

[b30] Laffont S., Siddiqui K. R. & Powrie F. Intestinal inflammation abrogates the tolerogenic properties of MLN CD103^+^ dendritic cells. Eur. J. Immunol. 40, 1877–1883 (2010). 20432234 10.1002/eji.200939957 PMC6108414

[b31] Roth R. & Mamula M. J. Trafficking of adoptively transferred B lymphocytes in B-lymphocyte-deficient mice. J. Exp. Biol. 200, 2057–2062 (1997). 9246787 10.1242/jeb.200.14.2057

[b32] Agenès F. & Freitas A. A. Transfer of small resting B cells into immunodeficient hosts results in the selection of a self-renewing activated B cell population. J. Exp. Med. 189, 319–329 (1999). 9892614 10.1084/jem.189.2.319 PMC2192996

[b33] Yamano T. . Thymic B cells are licensed to present self antigens for central tolerance induction. Immunity 42, 1048–1061 (2015). 26070482 10.1016/j.immuni.2015.05.013

[b34] Walters S. N., Webster K. E., Daley S. & Grey S. T. A role for intrathymic B cells in the generation of natural regulatory T cells. J. Immunol. 193, 170–176 (2014). 24872190 10.4049/jimmunol.1302519

[b35] João C., Ogle B. M. & Geyer S. Immunoglobulin promotes the diversity and the function of T cells. Eur. J. immunol. 36, 1718–1728 (2006). 16791877 10.1002/eji.200635908

[b36] João C., Ogle B. M., Gay-Rabinstein C., PLatt J. L. & Cascalho M. B cell-dependent TCR diversification. J. Immunol. 172, 4709–4716 (2004). 15067046 10.4049/jimmunol.172.8.4709

[b37] Noorchashm H. . B lymphocytes influence the shape of the mature preimmune CD4^+^ TCR repertoire. Transplant Proc. 31, 832–833 (1999). 10083361 10.1016/s0041-1345(98)01792-8

[b38] HayGlass K. T., Naides S. J., Benacerraf B. & Sy M. S. T cell development in B cell deficient mice. III. Restriction specificity of suppressor T cell factor(s) produced in mice treated chronically with rabbit anti-mouse mu chain antibody. J. Mol. Cell Immunol. 2, 107–117 (1985). 2978459

[b39] Barr T. A. . B cell depletion therapy ameliorates autoimmune disease through ablation of IL-6-producing B cells. J. Exp. Med. 209, 1001–1010 (2012). 22547654 10.1084/jem.20111675 PMC3348102

[b40] Molnarfi N. . MHC class II-dependent B cell APC function is required for induction of CNS autoimmunity independent of myelin-specific antibodies. J. Exp. Med. 210, 2921–2937 (2013). 24323356 10.1084/jem.20130699 PMC3865476

[b41] Yu S., Ellis J. S., Dunn R., Kehry M. R. & Braley-Mullen H. Transient depletion of B cells in young mice results in activation of regulatory T cells that inhibit development of autoimmune disease in adults. Int. Immunol. 24, 233–242 (2012). 22298883 10.1093/intimm/dxs003 PMC3312073

[b42] Mann M. K., Maresz K., Shriver L. P., Tan Y. & Dittel B. N. B cell regulation of CD4^+^CD25^+^ T regulatory cells and IL-10 via B7 is essential for recovery from experimental autoimmune encephalomyelitis. J. Immunol. 178, 3447–3456 (2007). 17339439 10.4049/jimmunol.178.6.3447

[b43] Matsushita T., Horikawa M., Iwata Y. & Tedder T. F. Regulatory B cells (B10 cells) and regulatory T cells have independent roles in controlling experimental autoimmune encephalomyelitis initiation and late-phase immunopathogenesis. J. Immunol. 185, 2240–2252 (2010). 20624940 10.4049/jimmunol.1001307 PMC3717968

[b44] Ray A., Basu S., Williams C. B., Salzman N. H. & Dittel B. N. A novel IL-10-independent regulatory role for B cells in suppressing autoimmunity by maintenance of regulatory T cells via GITR ligand. J. Immunol. 188, 3188–3198 (2012). 22368274 10.4049/jimmunol.1103354 PMC3311743

[b45] Yoshizaki A. . Regulatory B cells control T-cell autoimmunity through IL-21-dependent cognate interactions. Nature 491, 264–268 (2012). 23064231 10.1038/nature11501 PMC3493692

[b46] Maseda D. . Peritoneal cavity regulatory B cells (B10 cells) modulate IFN-γ^+^CD4^+^ T cell numbers during colitis development in mice. J. Immunol. 191, 2780–2795 (2013). 23918988 10.4049/jimmunol.1300649 PMC3770313

[b47] Schmidt E. G. W. . B cells exposed to enterobacterial components suppress development of experimental colitis. Inflamm. Bowel Dis. 18, 284–293 (2012). 21618359 10.1002/ibd.21769

[b48] Lampropoulou V. . TLR-activated B cells suppress T cell-mediated autoimmunity. J. Immunol. 180, 4763–4773 (2008). 18354200 10.4049/jimmunol.180.7.4763

[b49] Zelenay S. . Foxp3^+^CD25^−^ CD4 T cells constitute a reservoir of committed regulatory cells that regain CD25 expression upon homeostatic expansion. Proc. Natl. Acad. Sci. USA 102, 4091–4096 (2005). 15753306 10.1073/pnas.0408679102 PMC554795

[b50] Weiss J. M. . Neuropilin 1 is expressed on thymus-derived natural regulatory T cells, but not mucosa-generated induced Foxp3^+^ Treg cells. 2012. J. Exp. Med. 209, 1723–1742 (2012). 22966001 10.1084/jem.20120914 PMC3457733

[b51] Yadav M. . Neuropilin-1 distinguishes natural and inducible regulatory T cells among regulatory T cell subsets *in vivo*. J. Exp. Med. 209, 1713–1722 (2012). 22966003 10.1084/jem.20120822 PMC3457729

[b52] Zhou X. . Instability of the transcription factor Foxp3 leads to the generation of pathogenic memory T cells *in vivo*. Nat. Immunol. 10, 1000–1007 (2009). 19633673 10.1038/ni.1774 PMC2729804

[b53] Komatsu N. . Pathogenic conversion of Foxp3^+^ T cells into Th17 cells in autoimmune arthritis. Nat. Med. 20, 62–68 (2014). 24362934 10.1038/nm.3432

[b54] Zheng J., Liu Y., Lau Y. & Tu W. CD40-activated B cells are more potent than immature dendritic cells to induce and expand CD4^+^ regulatory T cells. Cell. Mol. Immunol. 7, 44–50 (2010). 20081875 10.1038/cmi.2009.103 PMC4003254

[b55] Morlacchi S., Soldani C., Viola A. & Sarukhan A. Self-antigen presentation by mouse B cells results in regulatory T-cell induction rather than anergy or clonal deletion. Blood 118, 984–991 (2011). 21652680 10.1182/blood-2011-02-336115

[b56] Nogueira J. S. . Enhanced renewal of regulatory T cells in relation to CD4^+^ conventional T lymphocytes in the peripheral compartment. Immunology 147, 221–239 (2016). 26572097 10.1111/imm.12555 PMC4717236

[b57] Almeida A. R., Zaragoza B. & Freitas A. A. Indexation as a novel mechanism of lymphocyte homeostasis: the number of CD4^+^CD25^+^ regulatory T cells is indexed to the number of IL-2-producing cells. J. Immunol. 177, 192–200 (2006). 16785514 10.4049/jimmunol.177.1.192

[b58] Almeida A. R., Rocha B., Freitas A. A. & Tanchot C. Homeostasis of T cell numbers: from thymus production to peripheral compartmentalization and the indexation of regulatory T cells. Semin. Immunol. 17, 239–49 (2005). 15826829 10.1016/j.smim.2005.02.002

[b59] Relland L. M. . The TCR repertoires of regulatory and conventional T cells specific for the same foreign antigen are distinct. J. Immunol. 189, 3566–3574 (2012). 22933635 10.4049/jimmunol.1102646 PMC3538134

[b60] Singh N. J., Bando J. K. & Schwartz R. H. Subsets of nonclonal neighboring CD4^+^ T cells specifically regulate the frequency of individual antigen-reactive T cells. Immunity 37, 735–746 (2012). 23021952 10.1016/j.immuni.2012.08.008 PMC3478444

[b61] Nishio J. . Requirement of full TCR repertoire for regulatory T cells to maintain intestinal homeostasis. Proc. Natl. Acad. Sci. USA 112, 12770–12775 (2015). 26420876 10.1073/pnas.1516617112 PMC4611614

[b62] Wang L. . T regulatory cells and B cells cooperate to form a regulatory loop that maintains gut homeostasis and suppresses dextran sulfate sodium-induced colitis. Mucosal Immunol. 8, 1297–1312 (2015). 25807185 10.1038/mi.2015.20 PMC4583327

[b63] Asseman C., Mauze S., Leach M. W., Coffman R. L. & Powrie F. An essential role for interleukin 10 in the function of regulatory T cells that inhibit intestinal inflammation. J. Exp. Med. 190, 995–1004 (1999). 10510089 10.1084/jem.190.7.995 PMC2195650

