# Supplementary material for: Enlarged colitogenic T cell population paradoxically supports colitis prevention through the B-lymphocyte-dependent peripheral generation of CD4+Foxp3+ Treg cells
Source: Sci Rep. 2016 Jun 29;6:28573. doi: 10.1038/srep28573 (PMC4926115; doi:10.1038/srep28573)
Supplement: Supplementary Information [file srep28573-s1.pdf]

Supplemental Data from the original manuscript by Canto *et al.* entitled **Enlarged colitogenic T cell population paradoxically supports colitis prevention through the B-lymphocyte-dependent peripheral generation of $CD4^+Foxp3^+$ Treg cells**

Fábio Barrozo do Canto, Sylvia M. N. Campos, Alessandra Granato, Rafael F. da Silva, Luciana Souza de Paiva, Alberto Nóbrega, Maria Bellio and Rita Fucs

a

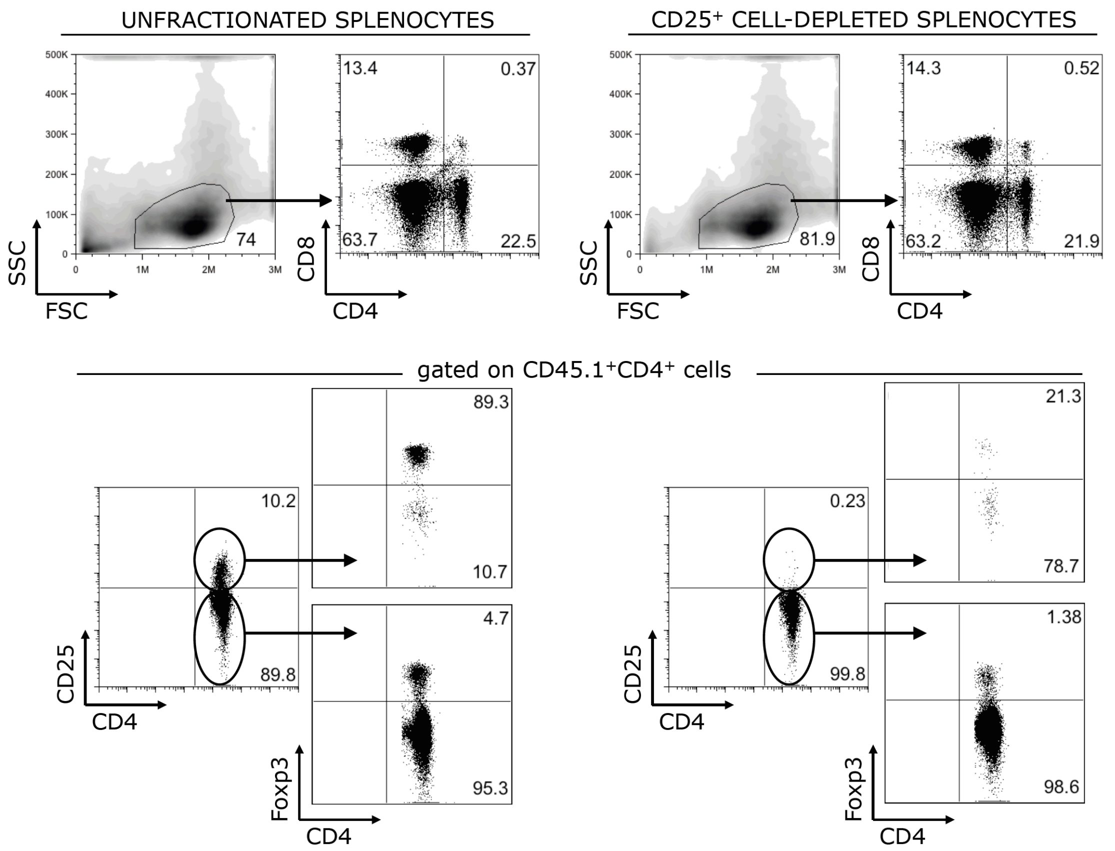

b

	Absolute numbers (x 10^6) of relevant T cell subsets present in the inocula				
	$CD4^+CD8^-$	$CD4^-CD8^+$	$CD4^+CD25^-$ $Foxp3^-$	$CD4^+CD25^-$ $Foxp3^+$	$CD4^+CD25^+$ $Foxp3^+$
Unfractionated (20 x 10^6)	3.32 ± 0.08	2.35 ± 0.38	2.87 ± 0.01	0.14 ± 0.004	0.27 ± 0.07
$CD25^-$ (20 x 10^6)	3.34 ± 0.16	2.23 ± 0.007	3.28 ± 0.14	0.046 ± 0.001	0.0025 ± 0.001
$CD25^-$ (40 x 10^6)	6.7 ± 0.32	4.6 ± 0.02	6.56 ± 0.31	0.092 ± 0.003	0.005 ± 0.003

Supplementary Figure 1: T cell frequencies are kept unaltered upon $CD25^+$ cell depletion and the absolute number of regulatory T cell contaminants in the inoculum is very low. Flow cytometric characterization of the inocula of unfractionated and CD25-depleted splenocytes. (A) Representative dot-plots show the percentages of CD4, CD8 and CD25 T cells in adult B6.SJL splenocytes before (unfractionated) and after depletion of $CD25^+$ cells by magnetic-activated cell sorting. As depicted, $CD25^+$ cell depletion was very

efficient (> 99.0% pure) and did not affect single-positive $CD4^+$ or $CD8^+$ T cell proportions. Intracellular staining for Foxp3 shows the relative percentages of Foxp3-expressing cells within $CD4^+CD25^+$ and $CD4^+CD25^-$ subsets prior to *in vivo* inoculation of either unfractionated or CD25-depleted spleen cells. (B) Data from three independent $CD25^+$ cell depletions are pooled and the absolute numbers of relevant T cell subsets (mean ± SEM) present in each type of inoculum are shown.

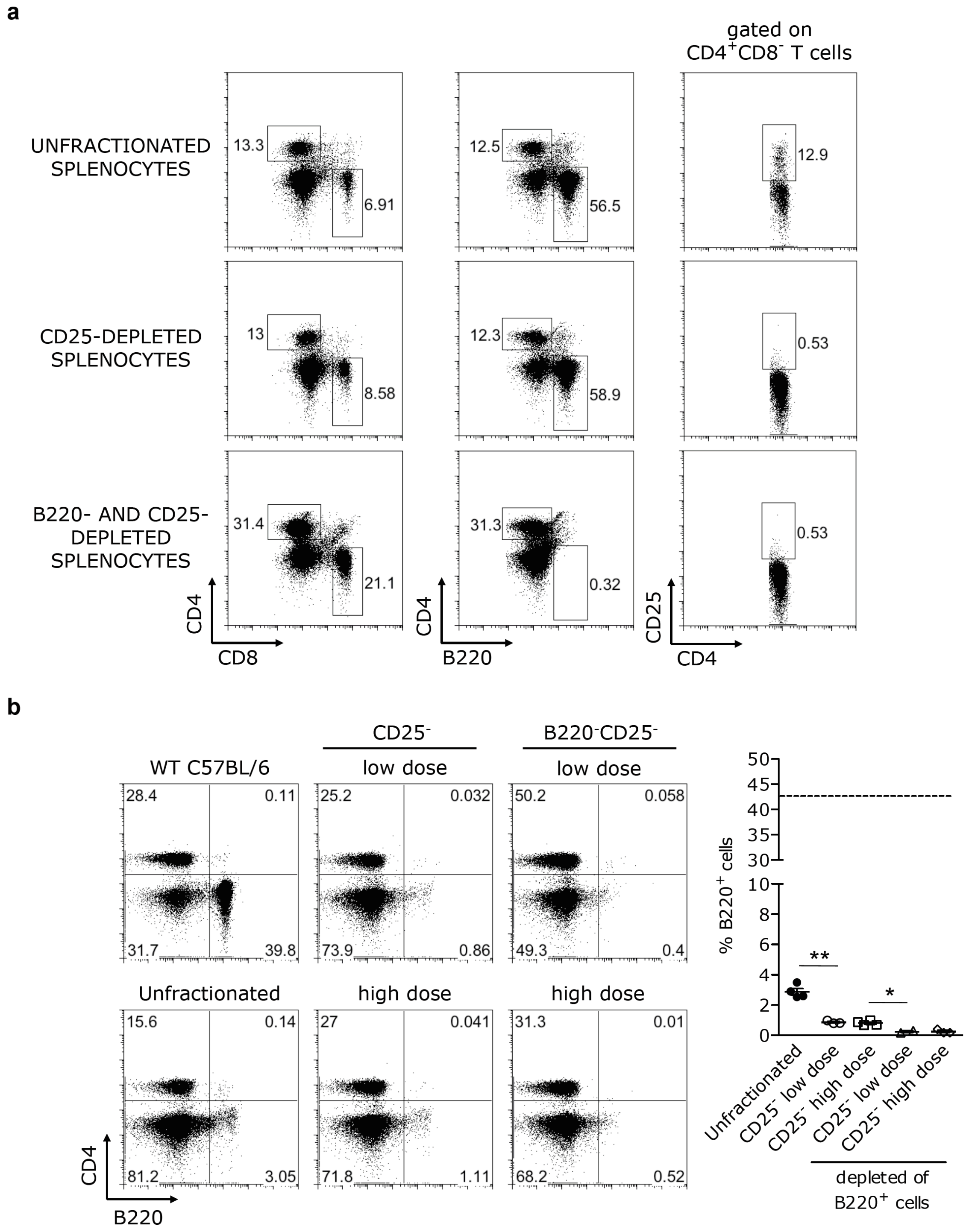

Supplementary Figure 2: B lymphocytes are scarcely found in the circulation of Rag$^{-/-}$ mice two weeks after reconstitution with B cell-containing splenocytes. (A) Splenocytes obtained from adult C57BL/6 donors were first depleted of CD25^{+} cells and then incubated with biotin-conjugated anti-B220 antibody for negative selection. B220^{-}CD25^{-} splenocytes were

approximatelly 99.5% pure, showing virtually no B220$^+$ or CD25$^+$ contaminants. The selective elimination of B cells, and not of other minor B220-expressing cell populations, was confirmed by checking the purity of the fraction attached to the magnetic collumn, which was more than 99.0% positive for the exclusive B-cell lineage marker CD19 (not shown). MACS-based elimination of B220$^+$ cells resulted in two-fold increase on the representation of CD4$^+$ and CD8$^+$ T cell subsets in B220$^-$ splenocytes. Accordingly, 10 and 20 x 10^6 of B220$^-$CD25$^-$ harbor about the same absolute numbers of CD4$^+$ and CD8$^+$ T lymphocytes present in low (20 x 10^6) and high (40 x 10^6) dose of CD25$^-$ splenocytes and, therefore, were the doses chosen for injection. **(B)** B6.*Rag2*$^{-/-}$ mice were adoptively transferred with either unfractionated splenocytes, splenocytes depleted of CD25$^+$ cells (20 or 40 x 10^6 cells per mouse) or splenocytes depleted of both CD25$^+$ and B220$^+$ cells (10 or 20 x 10^6 cells per mouse) obtained from adult C57BL/6 donors and, two weeks after injection, the frequencies of B220$^+$ cells were determined in blood by flow cytometry. Dot-plots representative of one animal within each experimental group are shown. Numbers plotted in each quadrant refer to the percentage of events accumulated on lymphocyte gate established on the basis of FSC x SSC parameters. Data including all experimental animals in each condition (with at least three mice per group, mean ± SEM) are shown. Average B220$^+$ cell frequencies found in euthymic B6 mice are included for comparison (dashed horizontal lines). *$p < 0.05$, **$p < 0.01$.

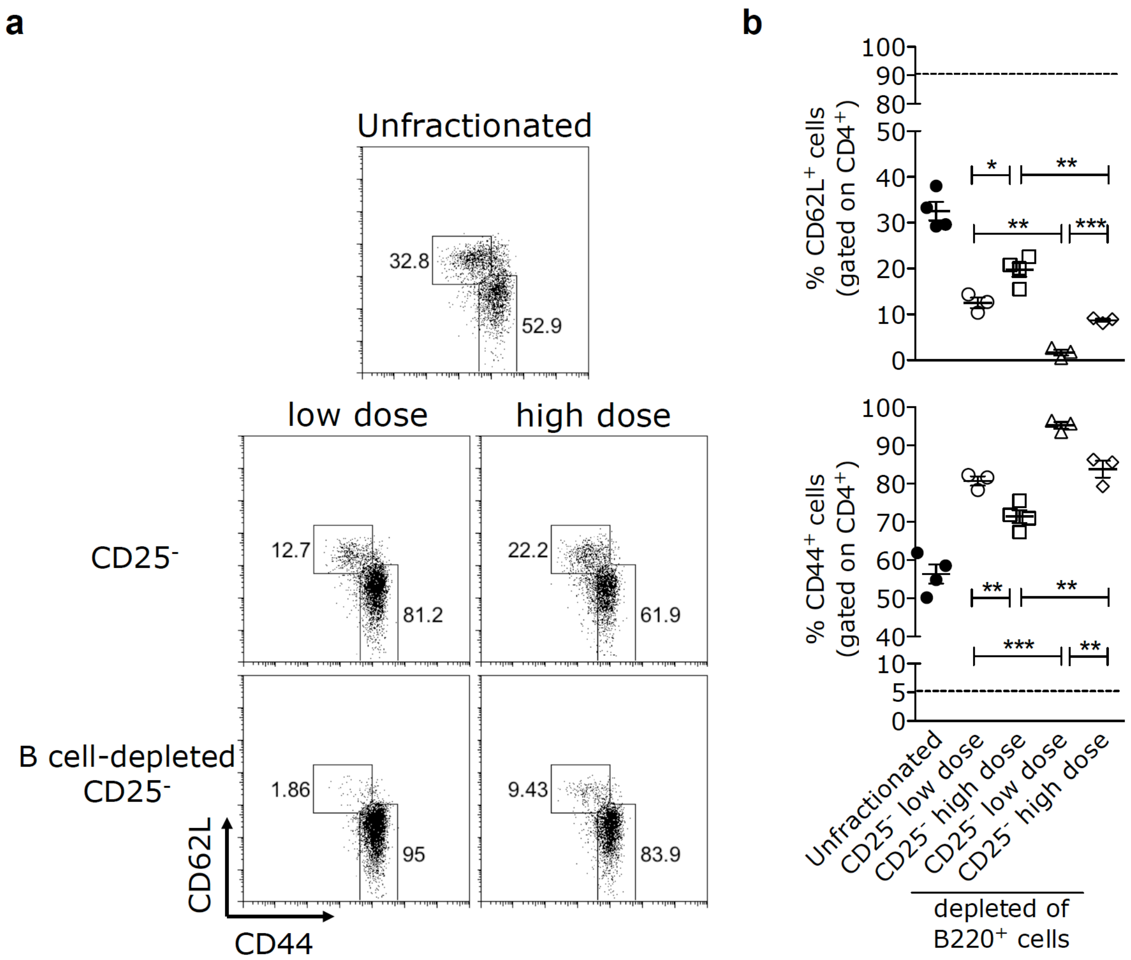

Supplementary Figure 3: B cells are required for the regulation of imbalanced CD62L/CD44 ratio within CD4$^+$ T compartment under lymphopenic settings. Blood samples of mice described in *Figure 4* were collected 28 days after adoptive transfer and the frequencies of effecttor-like CD44^{high}CD62L$^-$ and naïve CD44$^-$CD62L^{high} T cells were determined by flow cytometry. **(A)** Dot-plots representative of one animal within each experimental group are shown. Numbers plotted in each quadrant refer to the percentage of events accumulated on CD4$^+$CD8$^-$ T lymphocyte gate. **(B)** Data including all experimental animals in each condition (with at least three mice per group, mean ± SEM) show blood frequencies of CD62L$^+$CD44$^-$ (top) and CD44$^+$CD62L$^-$ (bottom) relative to total CD4$^+$ T cells. Average frequencies of those CD4$^+$ T cell

subsets in naïve adult B6 mice are included for comparison (dashed lines). *$p < 0.05$, **$p < 0.01$, ***$p < 0.001$; not significant (ns): $p > 0.05$.

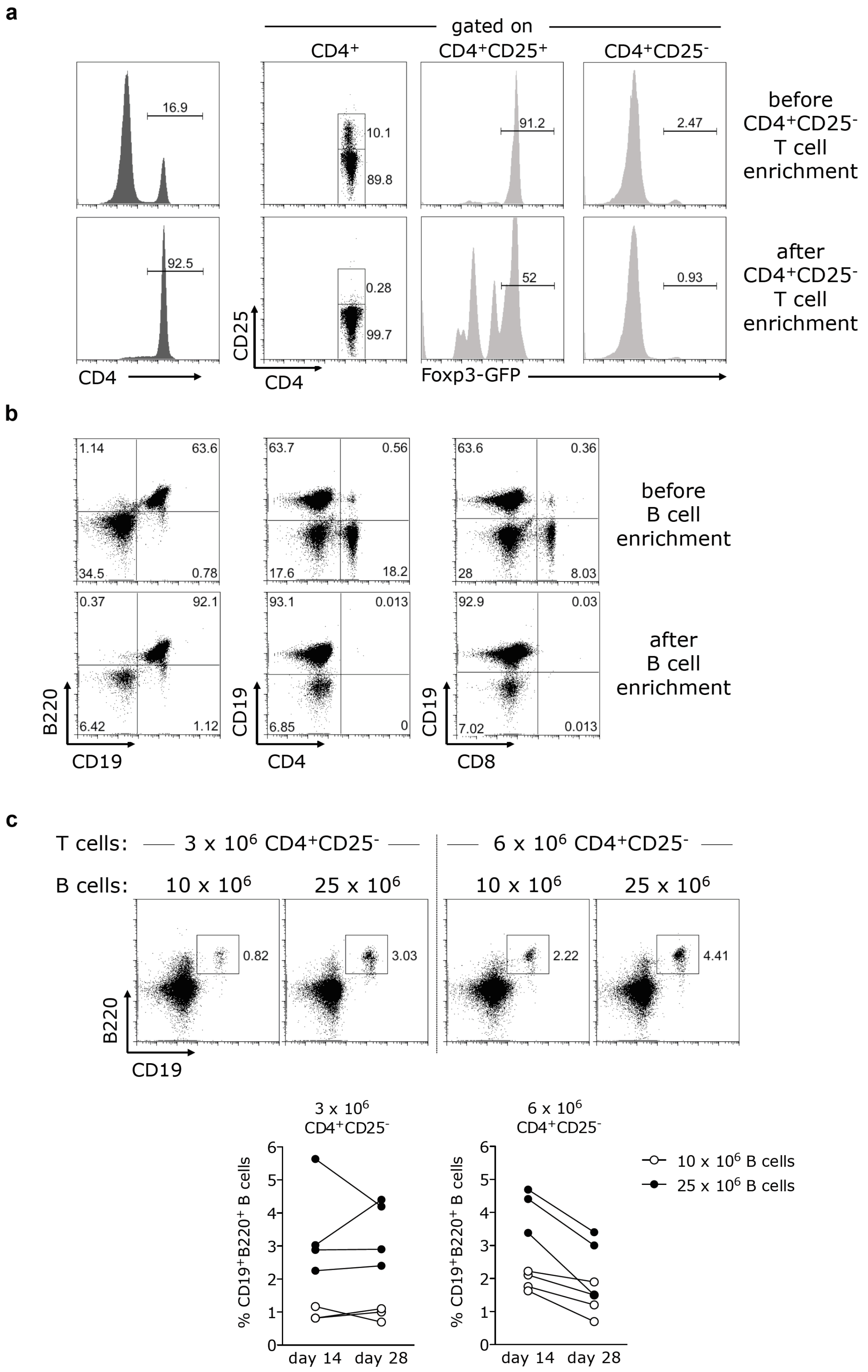
a
gated on
CD4+
CD4+CD25+
CD4+CD25-
16.9
10.1
89.8
91.2
2.47
before
CD4+CD25-
T cell
enrichment
92.5
0.28
99.7
52
0.93
after
CD4+CD25-
T cell
enrichment
CD4
CD25
CD4
Foxp3-GFP
b
1.14
63.6
34.5
0.78
63.7
0.56
17.6
18.2
63.6
0.36
28
8.03
before
B cell
enrichment
0.37
92.1
6.42
1.12
93.1
0.013
6.85
0
92.9
0.03
7.02
0.013
after
B cell
enrichment
B220
CD19
CD19
CD4
CD19
CD8
c
T cells: 3 x 10^6 CD4+CD25- 6 x 10^6 CD4+CD25-
B cells: 10 x 10^6 25 x 10^6 10 x 10^6 25 x 10^6
0.82
3.03
2.22
4.41
B220
CD19
3 x 10^6
CD4+CD25-
6 x 10^6
CD4+CD25-
% CD19+B220+ B cells
day 14 day 28
10 x 10^6 B cells
25 x 10^6 B cells

Supplementary Figure 4: Enrichment of $CD4^+$ or $B220^+$ cells by MACS-based negative selection. **(A)** Splenocytes isolated from adult C57BL/6.Foxp3-GFP donors were enriched for $CD4^+$ T cells accordingly to the protocol described in *Materials and Methods*. The flow-through was collected as an untouched $CD4^+CD25^-$ T cell-enriched fraction (purity > 92%) and the percentages of $CD25^+$ and $Foxp3^+$ (determined on the basis of GFP fluorescence) cell contaminants are shown. **(B)** Splenocytes isolated from adult C57BL/6 donors were stained with biotin-conjugated antibodies for B220, CD8, CD11b, CD11c, CD49d and Tγδ and then placed on LD collumn for negative selection. The flow-through was collected as an untouched B cell-enriched fraction. Splenocytes enriched for B lymphocytes cells were > 92% pure and included no CD4- or CD8-expressing cells. **(C)** Blood samples of mice described in *Figure 4* were collected 14 and 28 days after adoptive transfer and the frequencies of donor-derived $B220^+CD19^+$ B cells were determined by flow cytometry. Dot-plots representative of one animal within each experimental group show B cell frequencies 14 days after adoptive transfer. Numbers plotted in each quadrant refer to the percentage of events accumulated on lymphocyte gate established on the basis of FSC x SSC parameters. Data including all experimental animals in each condition (with at least three mice per group, mean ± SEM) show blood frequencies of $B220^+CD19^+$ B cells determined 28 days after injection.
